# Modelling Terrestrial and Marine Foraging Habitats in Breeding Audouin's Gulls *Larus audouinii*: Timing Matters

**DOI:** 10.1371/journal.pone.0120799

**Published:** 2015-04-14

**Authors:** Juan Bécares, Manuel García-Tarrasón, Dani Villero, Santiago Bateman, Lluís Jover, Víctor García-Matarranz, Carolina Sanpera, José Manuel Arcos

**Affiliations:** 1 Programa marino, Delegació de Catalunya, SEO/BirdLife, Barcelona, Spain; 2 Departament de Biologia Animal, Universitat de Barcelona, Barcelona, Spain; 3 Grup d’Ecologia del Paisatge, Àrea de Biodiversitat, Centre Tecnològic Forestal de Catalunya, Solsona, Spain; 4 Departament de Salut Pública, Facultat de Medicina, Universitat de Barcelona, Barcelona, Spain; 5 Dirección General del Medio Natural y Política Forestal, MAGRAMA, Madrid, Spain; 6 IRBio-Research Institute of Biodiversity, University of Barcelona, Barcelona, Spain; Thomas Jefferson University, UNITED STATES

## Abstract

Although the breeding ecology of Audouin’s gull has been widely studied, its spatial distribution patterns have received little attention. We assessed the foraging movements of 36 GPS-tracked adult Audouin’s gulls breeding at the Ebro Delta (NW Mediterranean), coinciding with the incubation period (May 2011). This also coincided with a trawling moratorium northwards from the colony. We modelled the distribution of the gulls by combining these tracking data with environmental variables (including fishing activities from Vessel Monitoring System, VMS), using Maxent. The modelling range included both marine and terrestrial areas. Models were produced separately for every 2h time interval across the day, and for 2 fishing activity scenarios (workdays *vs*. weekends), allowing to assess the spatio-temporal distribution patterns of the gulls and the degree of association with fisheries. During workdays, gull distribution at sea fully matched with fishing activities, both trawling (daylight) and purse-seining (nightime). Gulls tended to avoid the area under trawling moratorium, confirming the high influence of fisheries on the distribution patterns of this species. On weekends, gulls made lesser use of the sea and tended to increase the use of rice fields. Overall, Audouin’s gull activity was more intense during dailight hours, although birds also showed nocturnal activity, on both workdays and weekends. Nocturnal patterns at sea were more disperse during the latter, probably because these gulls are able to capture small pelagic fish at night in natural conditions, but tend to congregate around purse-seiners (which would enhance their foraging efficiency) in workdays. These results provide important insight for the management of this species. This is of particular relevance under the current scenario of European fisheries policies, since new regulations are aimed at eliminating discards, and this would likely influence Audouin’s gull populations.

## Introduction

Human activities have a strong impact on ecosystems, both marine and terrestrial [1]. Among them, the industrialisation of commercial fisheries and the proliferation of introduced species have become problems of global concern [2, 3]. Among many other elements of the ecosystem, seabirds have been notably influenced by these two activities [4, 5, 6, 7]. Indeed, fisheries have produced changes in abundance of natural prey for seabirds, often reducing significantly their availability [8]. At the same time, commercial fisheries have made available to seabirds large amounts of easily accessible and predictable food in the form of discards [9]. Although this anthropogenic food source can benefit seabird populations in the short term, it also carries counterparts such as lower quality of the food intake [10], higher levels of heavy pollutants associated to bottom-dwelling prey [11], changes in the seabird community in favour of the most opportunistic species [12] or, ultimately, a high fishing pressure that leads to overexploitation of natural prey [6, 13]. On the other hand, introduced species have affected seabirds in several ways, with predation at colonies probably being the most impacting [7]. The proliferation of some invasive species may also constitute a new food resource for predators, especially those with greater trophic plasticity [14].

The Western Mediterranean has been for centuries a highly humanised region, and as such, the local seabird community has been highly influenced by human activities, including fisheries and invasive species. This is the case of the coastal area of the eastern Iberian Peninsula. The region holds one of the most important fishing fleets in the Mediterranean, especially trawlers and purse-seine vessels, producing large amounts of discards that influence the biology of the local seabirds [13, 15]. On the other hand, since the 1980s, the introduced American crayfish *Procambarus clarkii* has colonized the adjacent coastal freshwater bodies in the area (mainly rice fields) [16, 17]]. This new resource has become very abundant in the rice fields of the Ebro Delta and the Albufera of Valencia, where it represents an important proportion of the diet for some species of the local bird community [18]. This great availability of abundant and predictable food resources, together with the protection of some potential breeding sites, allowed an increase of gulls and terns in the region [19]. Of particular relevance is the case of Audouin's Gull *Larus audouinii* an endemic species to the Mediterranean. This gull was regarded as one of the most scarce and endangered seabirds in the world in the 1970's [20], but has notably increased its population afterwards, becoming a common species in the Iberian Mediterranean coast, especially in the Ebro Delta [21, 22].

The feeding ecology of Audouin's Gull has been widely studied in NE Iberian waters. Fisheries and the resulting discards are one of the most important factors influencing Audouin's gull ecology [21]. Trawlers, which operate during the daylight hours, appear to be the most influencing fleet for these gulls, due to the high amounts of discards produced [23, 24]. However, Audouin’s gulls also associate with purse seine vessels, which operate at night targeting shoals of small pelagic fish. In this case, gulls take advantage from discards, but also feed on small epipelagic fish attracted to the surface by the lamps of the vessels [25, 26, 27]. According to isotopic studies, 39–48% of the diet of adult breeding Audouin’s gulls comes from demersal fish (and therefore from discards), 29–30% from small pelagic fish (which come from either discards, feeding associated to purse seines or natural feeding) and 20–31% from the American crayfish [28].

Although there are several studies linking the trophic and reproductive ecology of Audouin’s gull with fisheries, the study of the use of space has received little attention. Observational studies have allowed assessing changes in both the distribution range and the activity patterns of the species in relation to changes in fishing activity, but could not provide any detail on their spatiotemporal distribution patterns [29, 30, 31]. Further detail was provided by studies using remote tracking techniques, specifically radio tags [32] and satellite transmitters [33], but both types of devices are subject to relatively low precision and low frequency of signals, thus providing also limited detail.

In this paper we aim to overcome the methodological limitations of previous studies and to assess in detail the spatio-temporal distribution patterns of adult Audouin's Gulls during the breeding season, and their interaction with human activities, particularly fisheries, taking advantage of the developments on GPS tracking devices [34]. The study was conducted at the Ebro Delta breeding colony, and encompasses the analysis of the terrestrial and marine environments altogether, through generating habitat suitability models from GPS bird tracking data. These models intended to show the habitat use by the species, in presence or absence of fishing activities, taking into account the time of day. Results will contribute to the understanding of the ecology of this near threatened seabird, as well as its threats, and hence to the conservation policies of the species [35].

## Material and Methods

### Study Area

**Fig 1:**
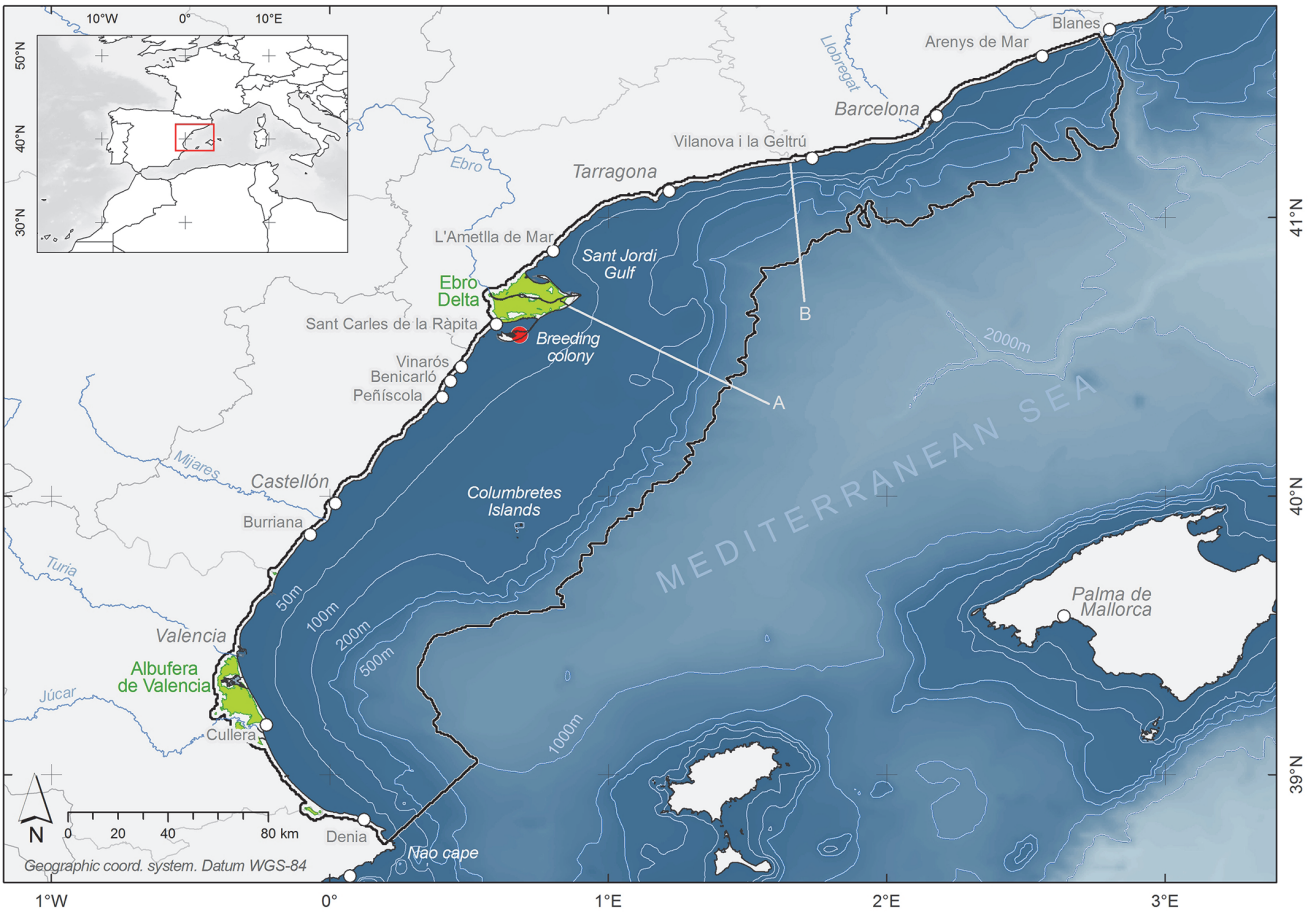
Study area. The red circle shows the Audouin’s gull breeding colony (Punta de la Banya, Ebro Delta) where the birds were trapped and GPS-tagged. The modelling area was defined by the movements of these gulls, and is shown bounded by a black line, covering both marine and terrestrial areas. Green areas indicate rice fields. The most important fishing ports are also shown (white circles). The area between lines A and B de fined the trawler moratorium area (see text for details).

The study area was defined from the movements of GPS-tracked Audouin’s gulls breeding at the *Punta de la Banya*, Ebro Delta, NW Spain (40°35’N, 0°40’E; S1 Fig), where the world largest colony of this near threatened species is located [22], holding about the 60% of the world population [36,37], 11,967 pairs in 2011 (Ebro Delta Natural Park *com*. *pers*.). The area comprised the Levantine coast of Spain, from *Cape de la Nao* (Alicante) to the *Tordera* river mouth (Barcelona), and extended from the coast over the continental shelf off to the upper slope (Fig 1). To encompass all GPS locations, the 1000 m isobath was used to define the offshore limit, whereas a 2 km band from the coast inland defined the terrestrial limit. This coastal band was extended further inland wherever rice fields were present (up to 11 km from the coast), since Audouin's gulls also exploit rice field paddies [38, 28, 39], and the GPS-tracked birds used them accordingly. In this way the study area comprised three main habitats: the sea, rice fields and fishing ports.

Marine waters in this area are highly productive within the Mediterranean context, influenced by strong prevalent winds from the Northwest, the Liguro-Provençal-Catalan shelf-slope current and the Ebro river inputs, as well as by the wide continental shelf that extends up to 70 km offshore [40, 41, 42]. This is reflected in the abundance of planktonic predators such as sardines (*Sardina pichardus*) or anchovies (*Engraulis encrasicolus*), which exploit the area to spawn [40]. These species are the main natural prey of Audouin's gull and other seabirds. This richness also supports an important fishing fleet, mostly trawlers (with 321 vessels within the study area) and purse-seiners (90 vessels) that operate locally across the whole continental shelf and slope, on a workday basis (from Monday to Friday) (Ministry of Food Agriculture and Environment, MAGRAMA *com*. *pers*.). Trawling is restricted to daylight hours (7:00–17:00h local time), while purse-seiners operate at night and dawn. The study period coincided with a trawling moratoria established from the Ebro river mouth northwards up to *Vilanova i la Geltrú* (Fig 1).

The coastline is highly urbanized in the study area, with few suitable foraging sites for gulls. Exceptions are some important wetlands, mainly the Ebro Delta and the Albufera de Valencia, and to a lesser extent the Llobregat Delta (Fig 1). The former two areas include large extensions of rice fields, with high densities of American crayfish [16]. Other sites include the numerous fishing ports scattered along the coast (Fig 1).

During the study period the weather remained calm and roughly uniform, with sunny sky and without strong winds.

### Audouin’s gull remote tracking

The study was conducted between 8th and 26th of May 2011 (data registered until 25th, S2 Fig), coinciding with the incubation period of the Audouin’s gulls. 60 adult breeding gulls were captured in the nest with either box or tent-labelled traps [43], and were then ringed, measured and equipped with CatTrack GPS loggers [44]. These loggers were programmed to collect locations every 5 minutes, which allowed for a battery life of 10–15 days. Accuracy is in most cases within the 10 m range [44]. The devices were sealed using a rubber shrink tube to ensure waterproof.

The GPS loggers were attached to the back of the gulls using a Teflon chest harness [34], to ensure that the birds could not tear off the devices. The harness had only two stitches to minimize the time of attachment in case that the birds could not be recaptured. The weight of the sealed devices plus the harness was 25g, roughly representing 3–5% of the bird’s body mass. Tagged birds were recaptured between one and two weeks after being tagged, using the same fieldwork procedure. Whenever appropriate, the traps used for the recapture were different to those used in the first capture, to minimize reluctance.

### Spatial patterns analyses

GPS locations were assigned to either foraging trips or colony locations. Only foraging locations were considered for modelling purposes. The foraging trip was defined as the locations since a bird leaves the colony until it returns [45]. For each GPS-location during the foraging trip, the speed was calculated and we estimated if the bird was resting (vel. < 1 km/h) or moving (vel. ≥ 1 km/h). The main habitats (sea, rice-field, and fishing port) were also assigned to each GPS-location. Spatial distribution models (SDM) were built by combining the foraging trip locations and habitat information (see details below). To account for differences in daily patterns and fishing activity, separate models were produced for different periods of the day, namely 12 time intervals (TI) of two hours each. Different models were also produced for workdays and weekends, to account for differences in fishing activity. Thus, 24 SDMs were finally conducted, accounting for 12 TIs in workdays and 12 TIs in weekends. TI followed the standard local time (summer time, GMT + 2).

#### Data filtering

For each TI, a single location per bird and day was selected at random from all the available data, both to ensure equal contribution by all individuals to the models and to reduce spatio-temporal autocorrelation [46, 47]. Moreover, all individuals used to build the models contributed with the same selected number of days (sND) to each SDM, although it could differ between models depending on data availability (which differed according to the type of day and the TI; S2 Table). Indeed, for each SDM we tried to find a compromise between sND used and the number of individuals reaching such sND to ensure a good sample size [48]. Thus, birds with ND ≥ sND were used to build the model (calibrate), whereas birds that did not reach sND (i.e. ND < sND) did not contribute to build the models, although they did contribute to the model validation process (see below). For workdays, sND used to calibrate the models varied from 1 to 4 days depending on TI (Table 1), while in weekends the information was more limited and sND was set at 1 in all cases.

**Table 1:** Audouin’s gull data selected for modelling (both for calibration and for validation), according to the time interval and the fishing activity (workdays vs. weekends).

	**Workdays**					**Weekend**				
**TI**	**NB**	**sNBc**	**sND**	**sLc**	**sNBv**	**NB**	**sNBc**	**sND**	**sLc**	**sNBv**
**00–02**	25	25	1	25	16	17	17	1	17	6
**02–04**	25	25	1	25	15	17	17	1	17	5
**04–06**	33	20	3	60	26	20	20	1	20	7
**06–08**	34	24	3	72	28	22	22	1	22	11
**08–10**	36	27	3	81	29	25	25	1	25	10
**10–12**	32	22	4	88	26	26	26	1	26	11
**12–14**	32	21	4	84	27	26	26	1	26	14
**14–16**	34	31	3	93	26	30	30	1	30	15
**16–18**	33	24	4	96	27	29	29	1	29	15
**18–20**	34	22	4	88	27	28	28	1	28	14
**20–22**	33	30	2	60	21	28	28	1	28	14
**22–24**	27	19	2	38	21	26	26	1	26	8

Number of birds (NB); selected number of birds to calibrate (sNBc); selected number of days (sND); selected locations to calibration (sLc); and selected number of birds to validate (sNBv). Only one location per bird, day and time interval (TI) was selected at random.

For the validation, only one location per bird and day was selected at random for each TI, both for workdays and weekends. Birds not used for the calibration (ND < sND) were used for validation. Furthermore, birds with ND > sND were also used to validate the model, excluding the days used previously to build the model (Table 1).

#### Environmental variables

To build the SDMs, we preliminarily selected those explanatory variables that could likely influence the distribution patterns of the Audouin’s gulls, based both on published information on the species and on modelling studies with other seabirds in the Mediterranean region [49, 31, 50, 51]. Differing to previous studies on spatial modelling of seabirds, which focused on the marine environment, here we combined information from both marine and terrestrial environments altogether, since Audouin’s gulls forage in both of them regularly [38, 29, 28]. The combination of these two environments with noticeable differences in available spatial resolution (SR) forced to find a compromise to define the working SR. This resolution was selected at 0.31 minutes of arc (‘), roughly equivalent to 500 m, using a geographic coordinate system in WGS-1984 datum. This resolution can differentiate well the land-sea border and terrestrial habitats. Working with this resolution forced to increase the pixel size for the terrestrial variables, as well as to refine the resolution for the marine variables (Table 2). Since Maxent cannot model in areas where one of the variables is *nodata* (see Modelling approach), and each environment had its own specific variables, we assigned arbitrary and meaningless values to marine variables in terrestrial areas, and the opposite was true for terrestrial variables in marine areas. Environmental variables are provided in Supporting Information (S3 Fig).

**Table 2:** Variables considered for the Audouin’s gull habitat modelling process.

**Variable**	**Units**	**oSR**	**TR**	**Source**
*Bathymetry*	meters	1’	-	ETOPO1 Global Relief Model [87]
Seafloor slope; Slope*	percentage	1’	-	Derived from ETOPO1 Global Relief Model [87]
Distance to the shoreline; CoastDist*	degrees	0.31’	-	Derived from GSHHS shoreline ^1^ [88]. Calculated as negative (land) and positive (sea)
Distance to the continent; ContinentDist*	degrees	0.31’	-	Derived from GSHHS shoreline ^1^ [88]. Calculated as negative (land) and positive (sea)
Altitude above sea level; *MASL*	meters	30m	-	Derived from ASTER GDEM [89]
Rice fields cover; RiceFieldsCov*	percentage	100m	-	From Corine Land Cover 2006 (level 3) [90]. Downloaded from Centro Nacional de Información Geográfica (CNIG)^2^.
Rice fields altitude; *RiceFieldsMASL*	meters	30m	-	Derived from ASTER GDEM [89]
Distance to the breeding colony; *ColonyDist*	degrees	0.31’	2011	Calculated as cost distance (*Cost distance tool*, ArcGis)
Fishing ports cover; *PortsCov*	percentage	200m	-	From BCN200 (Spanish National Base Cartographic) of CNIG ^2^
Distance to the active trawling ports TrawPortDist*	degrees	0.31’	-	Calculated as cost distance (*Cost distance* tool, ArcGis). Information about fishing ports from MAGRAMA [91] *Trawling ports in trawling moratorium areas are not considered*.
Distance to the purse seine ports PurSePortDist*	degrees	0.31’	-	Calculated as cost distance (*Cost distance* tool, ArcGis). Information about fishing ports from MAGRAMA [91]
Distance to both trawling & purse seine ports; *PortsDist*	degrees	0.31’	-	Calculated as cost distance (*Cost distance* tool, ArcGis). Information about fishing ports from MAGRAMA [91]
Chlorophyll concentration; *chl2011may*	mg/m^3^	1km	Daily	From Aqua-Modis (level 2) [92]. Averaged for the days 8 to 25 May 2011
Sea surface temperature (SST); *sst2011may*	brightness temperature	1km	Daily	From Aqua-Modis (level 2) [92]. Averaged for the days 15 to 17 May 2011
Purse seine density (estimated separately for each TI)	VMS locations /km^2^	0.31’	≤ 2h (8–25 may)	Purse seine VMS data supplied by *Centro de Seguimiento de Pesca (MAGRAMA)*. Calculated by *Point Density tool* (ArcGis)
Trawlers density (estimated separately for each TI)	VMS locations/km^2^	0.31’	≤ 2h (8–25 may)	Trawlers VMS data supplied by *Centro de Seguimiento de Pesca (MAGRAMA)*. Calculated by *Point Density tool* (ArcGis)

The original spatial resolution (oSR), the temporal resolution (TR), and the source of the variables are shown. Those variables with an asterisk (*) were eventually not used to build the models (see text for details).

^1^ http://www.ngdc.noaa.gov/mgg/shorelines/shorelines.html

^2^ http://centrodedescargas.cnig.es/CentroDescargas/buscadorCatalogo.do

The marine variables selected were bathymetry (sea depth), slope, chlorophyll concentration (Chl-a), sea surface temperature (SST), and densities of both trawlers and purse-seiners for each time interval. The *bathymetry* and *slope* are related to the marine topography and subsequently with oceanographic circulation, productivity and prey distribution [42]. Chl-a was taken as a proxy of productivity, and hence of food availability, while SST can also influence primary production [52, 51]. We used the average of daily data of both variables (for more details see S1 Text), and were called *chl2011may* and *sst2011may*. Information on the distribution of fishing vessels was available from the Vessel Monitoring System (VMS), which was provided by the Spanish Ministry of Environment (MAGRAMA). This system collects a vessel GPS position every two hours, though it only works for fishing vessels larger than 15m long. All VMS data within the study area coinciding with the study period were incorporated to the models considering separately trawlers and purse-seiners. These data correspond to a 92.2% (n = 83) purse-seine vessels present in the ports of the study area and a 90.1% (n = 221) of active trawlers (in ports without moratorium). Other fishing practices in the area are mostly conducted by smaller vessels of artisanal type that do not provide VMS information, and thus were not considered here. Nevertheless, trawlers and purse-seiners are the vessels that most frequently attract seabirds in the area [26]. Densities of both trawlers and purse-seiners were calculated for the different TIs when these vessels operate: 6 TI from 22:00 to 10:00h in the case of purse-seiners (*PurSeDe*), and 6 TI from 06:00h to 18:00h in the case of trawlers (*TrawDe*). To calculate these densities, only one location per vessel, day and TI was selected, at random.

The terrestrial variables selected were altitude above sea level (*MASL*), fishing ports cover, rice fields cover and rice fields altitude. *MASL* was taken as the topographic variable on land. Fishing ports often congregate Audouin’s gulls, so that fishing ports cover (*PortsCov*) was created (this variable includes inner port waters). To convert this categorical variable into a continuous variable, we calculated the coating of port per pixel (0 to 1). This was done because the original SR is greater than the working one (Table 2; for more details see S1 Text). Another inland habitat used by the Audouin gulls is rice fields [38, 28], and rice fields cover (*RiceFieldsCov*) was thus added to the list of variables. It was calculated using the same procedure as with *PortsCov*. *MASL* of rice fields (*RiceFieldsMASL*) was considered as a proxy of soil salinity, and hence of crayfish presence or abundance probability since crayfish do not tolerate saline soils, which occur in lowlands, even if rice is cultivated there by flooding the fields with fresh water [53]. To create this variable we cut *MASL* using a mask of rice use; an arbitrary value of -1 was assigned to the rest of the study area (both land and sea).

Distance variables were created for both the marine and terrestrial environment, and were calculated as Cost Distance in ArcGis, restricting the passage only to the study area. Distance to the coast (*CoastDist*; either island or continental coastline) and only to continent (*ContinentDist*) were considered and calculated as positive values for locations at sea and negative for locations on land. We also calculated the distance to the breeding colony where birds were marked (*ColonyDist)*. Distance to fishing ports was considered because of Audouin’s gulls often follow fishing vessels to take profit of their discards when they come back to port [54, 31]. Hence we computed the distance to fishing ports, differentiating between trawling ports (*TrawPortDist*), purse-seining ports (*PurSePortDist*) and both trawling and purse-seining ports (*PortsDist*).

Finally, to avoid the excessive collinearity between variables included in each model, we conducted a Spearman correlation matrix in R software, and for each TI we excluded highly correlated variables; | rs | ≥ 0.70 (S4 Table and S2 Text). This reduced relevant variables from 16 to 10 to feed the modelling process.

#### Modelling approach

We used a modelling method based on maximum entropy implemented in the software MAXENT [55], because of its flexibility when handling different types of data and responses. This revealed as one of the most practical methods for modelling species distributions, which allows working only with presence data [56]. Hence it is particularly useful to deal with tracking data, which only provides positive information (i.e. presence-only data) [57]. The models were calibrated constraining the response of environmental variables to linear and quadratic functions, due to the difficulty of interpreting more complex relationships, and because these are the functions that better reflect the response of the species to the environment [58]. A setting *hinge feature* in Maxent was also added, allowing to identify turning points in the linear or quadratic response settings [59]. This adjustment was made due to the complexity of using at the same time variables at both sea and land, with an abrupt change in the biological significance between two environments (see environmental variables).

We performed SDMs for the 12 daily TIs, separately for workdays and weekends, thus producing 24 final SDMs. Data selected for calibration and validation were far lower than the total data available (we recorded up to 24 locations for each TI, day and bird), and hence potentially relevant information could have been missed. To minimize related biases, all the filtering and model building procedure was repeated 10 times per TI and fishing situation (workday and weekend), independently [57]. Thus we obtained 10 replicates for each TI and fishing situation, which were averaged to provide a single output map. Mapping units obtained correspond to a habitat quality index comprising values between 0 (minimum) and 1 (maximum).

#### Model evaluation

We tested the predictive ability of the SDMs using cross-validation with data that were not used in model calibration to avoid autocorrelation (see details above) [60]. Validation samples used represented an average of 31.4% data (SD = 5.8, range 21.9–45.7). The predictive reliability of the models was evaluated with the AUC statistic (Area Under the Curve). This statistic measures the area under the ROC (Receiver Operating Characteristics) curve, which provides a measure of the models predictive capability ranging from 0.5 (no predictive power) to 1 (a perfect model) [61, 62, 63]. To test the differences between the models generated on weekends and on workdays, an AUC cross validation (cvAUC) of samples between workdays and weekends was conducted.

### Ethic statement

All work was carried out with the necessary permits, awarded by both the Wildlife Service of the Catalan Government and the Ebro Delta Natural Park. A detailed description and justification of the fieldwork procedures was presented to these institutions, including the use of harnesses and timeliness of device removal, to get these permits. Handling time was minimized to reduce any inconvenience to the birds, not exceeding 10 minutes in any case. Birds were captured in daylight hours, avoiding periods when either low (dawn) or high temperatures (noon) could pose a problem for the eggs. Whenever a bird was reluctant to get captured, we left it. The tags represented 3–5% of the weight of the birds; about the recommended limit [64, 65, 66].

## Results

Of 60 Audouin’s gulls tagged, GPS data from 36 individuals were obtained, providing 9.6 ± 5.5 days of data (median ± IQR) (range 0.8–15.6 days) (more details in S1 Table). The remaining birds either could not be recaptured (n = 20) or presented GPS sealing failure and did not provide any data (n = 4). In total, 89,800 locations were obtained, of which 38,090 corresponded to foraging trips. Of the latter, 28,844 locations corresponded to workdays, and 9,246 to weekends.

### Movements and activity patterns

**Fig 2:**
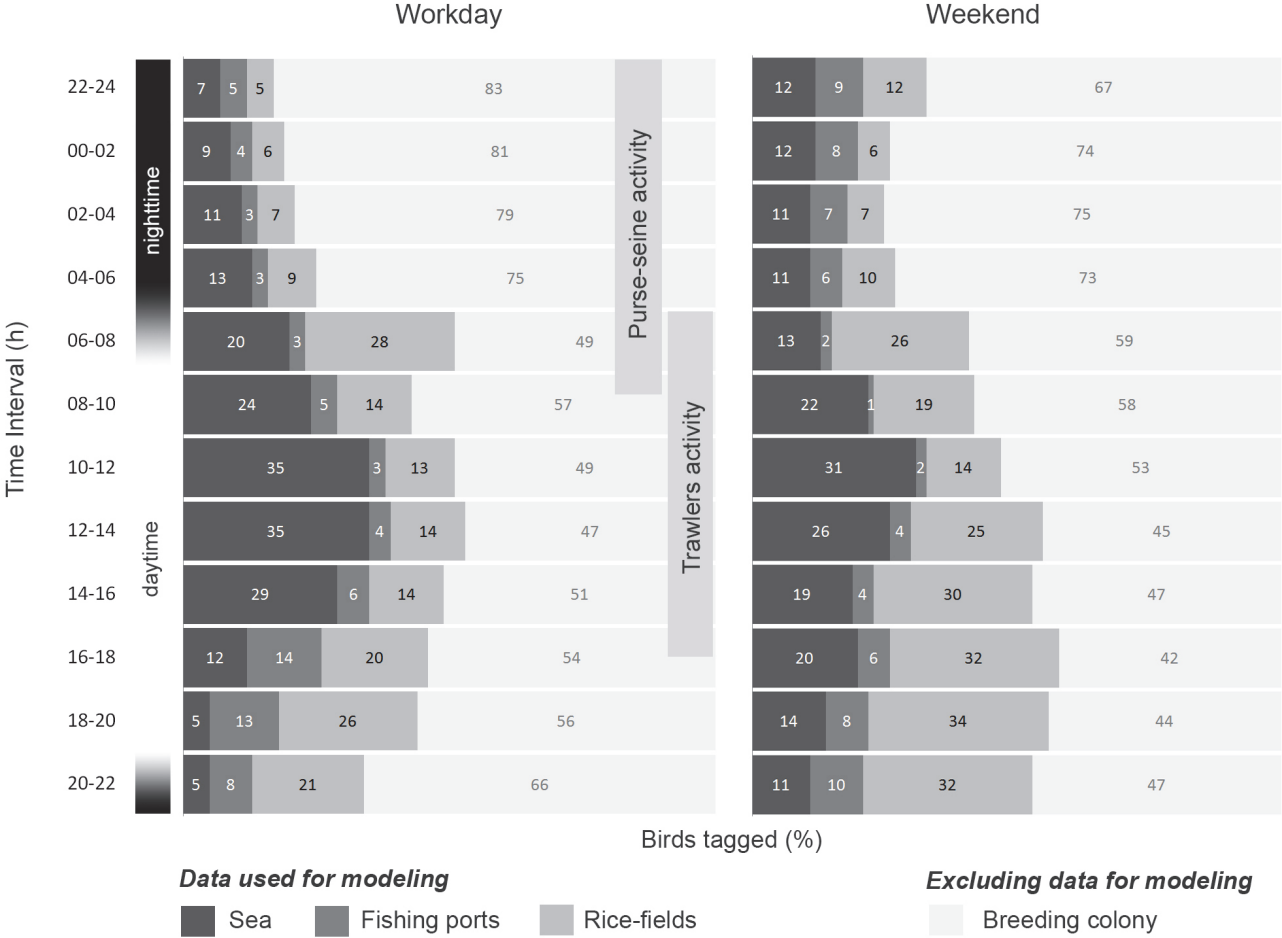
Habitat distribution of tagged Audouin’s gulls. A percentage of locations at sea, fishing ports, rice-fields or in the breeding colony are showed for each time interval, for workdays and weekends. Trawlers and purse-seine activity are indicated.

**Fig 3:**
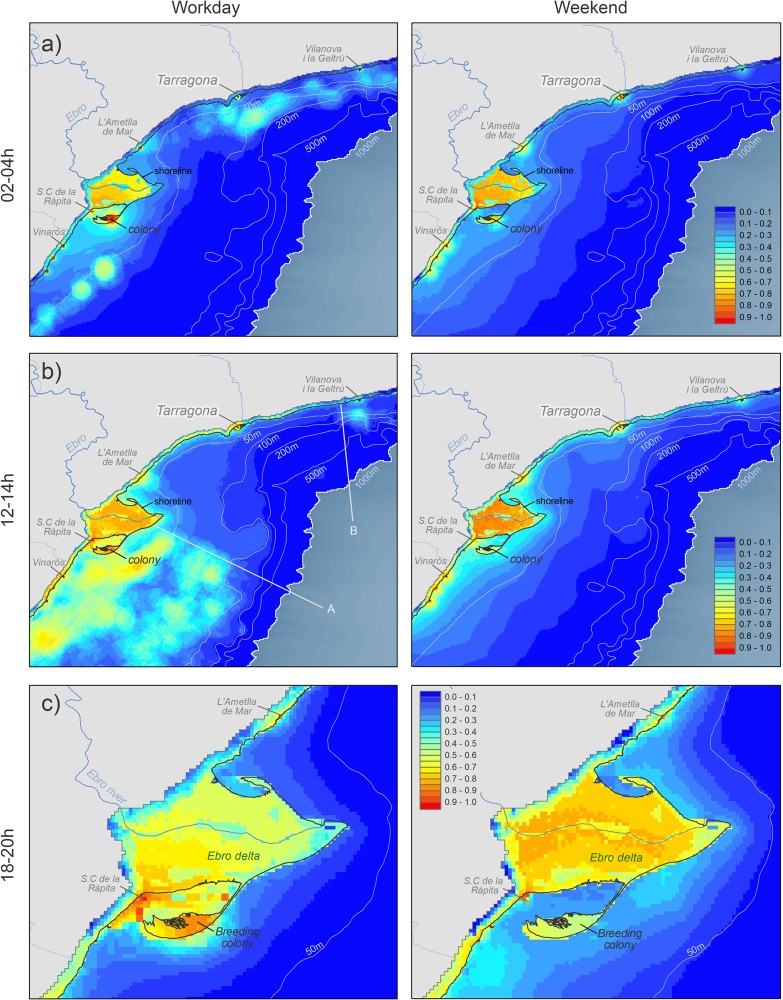
AUCs values for workday SDMs resulting from the validation of the models with the sample reserved for validation. Median (lines) and interquartile ranges (box plots) are shown. Blue boxes show the AUC for cross-validation (cvAUC) between workdays and weekends for each time interval.

Foraging trips had duration of 8.9 ± 6.14 hours (median ± IQR) (range 0.5–77.3 hours) (S4 Fig). Audouin’s gulls were mostly diurnal, although birds were also active at night (Fig 2). Birds spent almost half of the daylight hours outside the colony (49.8%, SD = 11.1). At night the activity out of the colony was reduced to about a half (25.8%, SD = 20.5) and in some birds was negligible (14.3% of individuals spent less than 5% of nighttime hours outside the colony). Most of the time spent outside the colony corresponded to resting behavior (50.5%, SD = 14.01). Bird’s activity was markedly different across environments: they were mostly resting in ports (84.0% of the time, SD = 11.5), about half of the time in rice fields (53.0%, SD = 17.5) and over a quarter of the time at sea (25.16%, SD = 12.6). At sea, the gulls mainly used the continental shelf waters south of the Ebro Delta, largely avoiding areas subject to trawl fishing moratorium (Fig 3). Considering data for working days between 10 and 16h (peak trawling activity), the Audouin’s gull density was more than 6 times higher in trawling areas than in areas under moratorium. All birds went directly out to the sea, while most often followed the coastline in their return trips. Many rested in the main fishing ports or in other breeding colonies (Castellón and Llobregat Delta) before returning to the colony. Although birds mainly visited neighboring rice fields at the Ebro Delta, two of the individuals were recorded to feed and to rest in the rice fields of the Albufera de Valencia, about 150 km southward from the study colony (Fig 1).

### Species Distribution Models (SDM)

**Fig 4:**
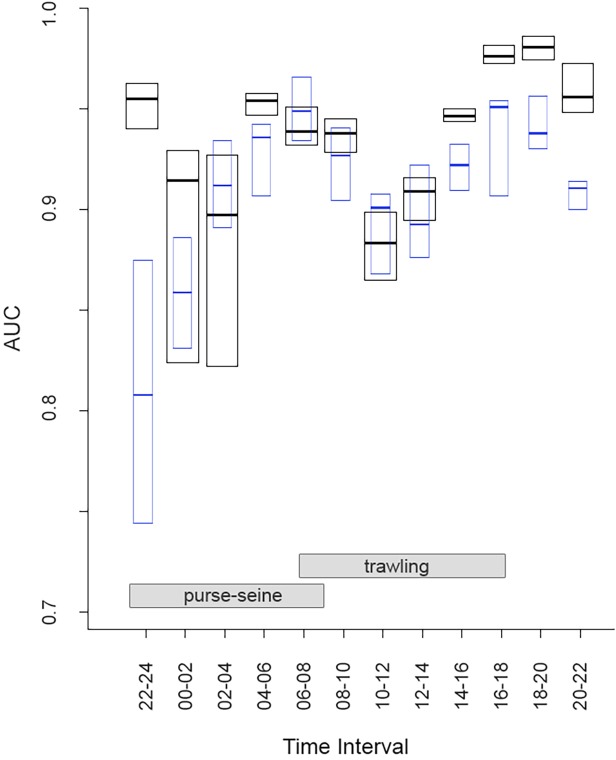
Some examples of Audouin’s gull distribution models for both workdays and weekends. a) purse-seine activity (TI: 02–04h), b) trawling activity (TI:12–14h; A and B lines delimited the area under trawler moratorium) and c) no fishing activity (TI: 18–20h; a coarser scale was selected here, as gull distribution at sea was marginal at this time interval, and focusing on the Ebro Delta allowed to better show the differences between working days and weekends).

All models generated (n = 240) showed a high predictive power (workdays AUC: mean = 0.93, SD = 0.04, range 0.76–0.99; weekends AUC: mean = 0.90, SD = 0.06, range 0.67–0.97; Fig 4). The best results (AUC> 0.90) and therefore with an excellent level of prediction were obtained in TI with a larger validation sample (mainly during daylight hours and in workdays). For TI in workdays (tested with weekends) between 14:00 and 24:00, the cvAUC showed significant statistical differences (one factor Anova test p<0.05; Fig 4).

Three representative time intervals were selected to illustrate the Audouin's gull SDMs (Fig 3). Between 02–04h (nighttime) in relation to purse-seine activity (Fig 3A), time interval 12–14h (daylight) associated to trawling activity (Fig 3B), and finally 18–20h (daylight), a period without fishing activity, in this case focusing in the Ebro Delta rice fields since suitability indexes at sea are very low (Fig 3C). In all cases the activity is compared between workdays (fishing activity) and weekends (without fishing activity). Figures corresponding to 24 TIs are provided in Supporting Information (S5 Fig).

Activity of individuals at nightime (25.8%) took place mainly in the rice fields or at sea (Figs 2 and 3A). On workdays birds concentrated their activity at sea during the study period in areas around 50m depth, coinciding with areas of high intensity of purse-seine activity. It is remarkable that on weekends night activity at sea was similar to workdays, although there is not purse-seine activity. In these cases birds distributed at sea between the coast and 50–75m depth or concentrated near the main fishing ports (Figs 2 and 3A). Activity observed on rice fields at night was similar between workdays and weekends.

During the morning and the central hours of the workdays (10–16h), the SMDs showed the better suitability indexes at sea, and the Audouin’s gull activity at sea was maximum, while on weekend’s suitability indexes and activity was lower (Figs 2 and 3B). By contrast, the activity in the rice fields increased during the weekend. At sea the gulls showed a widespread distribution over most of the continental shelf off the Ebro Delta, but they avoid the marine area under trawling moratorium (Fig 3B). In subsequent hours (16–18h), when trawlers return to the port, gull abundance at sea was reduced (Fig 2), and better suitability indexes at sea were close to the coast, especially near main fishing ports (S5 Fig). After 18h, Audouin’s gulls were not at sea on workdays. Normally, at this time we observed birds returning to the colony or tend to concentrate in fishing ports and rice fields (Figs 2 and 3C).

**Fig 5:**
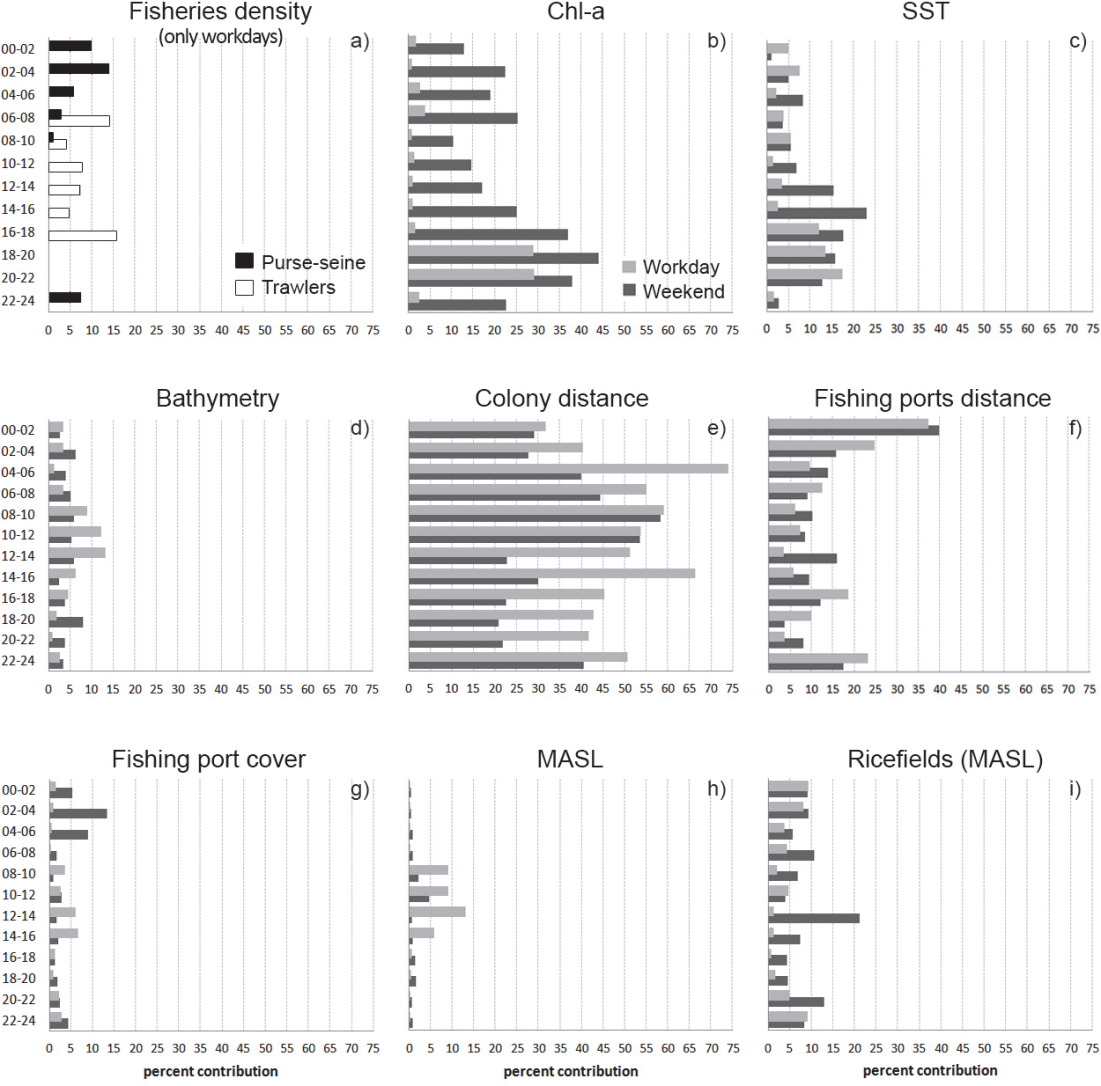
Variables percent contribution by time interval. Contribution of both purse-seine and trawlers (a) are showed in the same picture (only for workdays). For all other variables (b-i) the percent contribution for workdays and weekends are showed jointly.

**Fig 6:**
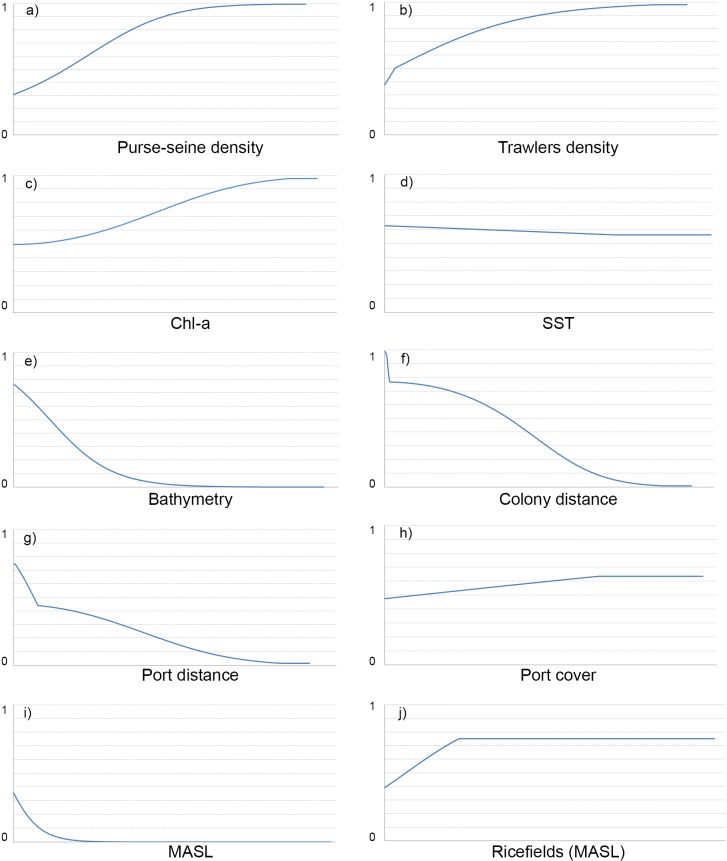
Response curves relating the probability of presence (0–1) of Audouin’s gull. Higher values correspond to higher probability of presence. The curves show how the logistic prediction changes as each environmental variable is varied, keeping all other environmental variables at their average sample value. The figure shows only a representative response curve for all time intervals, both for workdays and for weekends.

The daily pattern at this time differs in the weekends, when birds made a far lower use of the open sea, but tended to show some activity both during the central hours of the day and also in the late afternoon. In this case the cells with the best suitability indexes at sea were relatively near the coast, in depths around 50m and both north and south of the breeding colony, independently of the moratorium area, and also in areas with high Chl-a concentration (Fig 3B). Rice fields were more important during weekends (Fig 5), especially from noon until night (12–24h, Fig 2), and rice fields situated above 4 m.a.s.l. showed a better suitability index than those located at lower altitudes (Fig 6J).

The variables that most contributed to the SDMs were *ColonyDist*, *PortsDist* (for both further distance implies lower probability of presence; Fig 6F and 6G), *Chl-a* and SST (Fig 5). *ColonyDist* is for most time intervals, the variable with a higher contribution. This variable has a similar response in workdays and weekends during nighttime and until noon. However, after noon *ColonyDist* have a lesser contribution in weekends than in workdays (Fig 5E). *PortsDist* is important, especially at nighttime (specifically between 22–04h) in both weekends and workdays. Variables related to productivity, especially *Chl-a*, showed an important contribution to the model, especially on the afternoon and the nighttime during weekends (Fig 5B). *Bathymetry* did not show an outstanding contribution, probably because other variables related to productivity (*Chl-a* and *SST*) or *PortsDist* (closely related to the distance to shoreline), masks its effect. In TIs with fishing activity, both *PurSeDe* and *TrawDe* take important values of contribution (Fig 5A), higher vessel density implies a greater probability of presence (Fig 6A and 6B). Those variables exclusively terrestrial have a lower contribution to the overall model, probably because these habitats occupy a small percentage of the total study area. In that case the variable with more influence on Audouin’s gull distribution is *RiceFieldsMASL*, especially on weekends and highlighting from noon and during all afternoon on weekends (Figs 3C and 5I), followed by *PortsCov* that is relevant (despite its small size) during weekend night hours (Fig 5G).

## Discussion and Conclusions

The present work is the most accurate spatio-temporal study carried out in Audouin’s gull so far, thanks to: (1) the high precision and frequency of GPS data; (2) the use of detailed information on fishing activity (VMS system); (3) the joint modelling of both marine and terrestrial environments; (4) the consideration of different time intervals and different fishing situations (workdays vs. weekends); (5) the implementation of a trawling moratoria in half of the study area; and (6) the relatively large number of birds tracked. Indeed, all these factors together allowed accurately assessing the distribution and activity patterns of adult breeding Audouin’s gulls under different situations, providing insight on how different factors influence the ecology of this near threatened seabird. As far as we know, the combination of marine and terrestrial environmental variables in the same modelling area had not been addressed before. This is important to get a complete view of the ecology of many gulls and terns, since they commonly use both environments extensively, although studies are usually focused on only one of them. On the other hand, this could also reduce the accuracy of the model distribution results in both environments, masking information that might be relevant, but providing an overview of high interest. In the case of Audouin’s gull, only two works considered before the marine and freshwater distribution simultaneously [29, 33]. However, the methodologies used in these studies present some spatial and temporal limitations (see Introduction) and they did not apply any modelling methodology.

The study also confirms the wide foraging range and foraging flexibility of Audouin’s gulls, with trips lasting up to 3 days (77.3 hours), alternating different daily periods and habitats, and extending up to 190 km from the colony. This is quite an extreme strategy for a gull, since in most species trip duration does not exceed a few hours and is restricted to a few 10s of km [67, 68].

### Fisheries define spatio-temporal Audouin’s gull distribution at sea and on land

During trawling activity (8:00h to 18:00h), at sea activity of Audouin’s gulls was higher on weekdays than on weekends, fully matching with the distribution of trawlers. Moreover, the gulls tended to avoid the area under trawling moratorium. This confirms the great influence of trawling discards, an easily accessible and predictable food resource, on the ecology of this species. In fact, some authors have estimated that trawling discards account for more than 75% of the energy required by breeding Audouin's gulls and represent the bulk of their marine diet [23, 69]. When trawling activity finishes (18:00h), gulls head towards the fishing ports and towards the colony after having met their energy requirements. Afterwards, the probability of finding gulls at sea is very low.

Regarding workday nights, purse-seine activity appeared to define the spatio-temporal distribution of the gulls at sea. Both gulls and purse-seiners concentrated during the study around the 50 m depth isobath, where these vessels most often target sardines. During this association, gulls are fishing by themselves (taking advantage of the vessel light) or exploiting the purse-seine discards [26]. However, during the weekend’s night-time (when purse-seiners do not operate) the gull activity at sea was similar-or even higher- than on workdays, although birds dispersed more widely (up to 75–100 m depth). These best suitability areas match with high productive areas, as shows the contribution of the variable *Chl-a*. Probably, almost full moon and clear sky conditions during the study period would facilitate the natural fishing in this species, adapted to the capture of Clupeiform fish at night [70, 71, 72]. This is also supported by the greater activity observed both, at sea and in rice fields during the moonlight period, especially obvious for the first weekend of data, which comprised the 84.4% of total weekend data (moonlight from dusk until 04:40 [73]; S3 Table).

Fishing activity also appeared to influence the distribution of Audouin's gulls in neighbouring rice fields. It is especially remarkable that on weekends the rice field’s suitability index and the activity patterns were significantly higher than on workdays, especially from noon until midnight (12–24h), probably due to the food shortage caused by the absence of fishing activities. This suggests that this habitat is of secondary importance to the gulls, although the American crayfish represent an abundant and accessible food resource, closer to the colony than most fishing vessels. The fact that rice fields showed higher suitability indexes that the sea is probably due to the far smaller area occupied by this resource compared to the marine environment, thus concentrating all the gulls using this habitat in a very limited extension. Moreover, Audouin’s gulls spend more time resting at rice fields than at sea. It is noteworthy that rice fields below 4 meters above sea level showed lower suitability index than rice fields located at higher altitudes. This is probably related to the higher salinity of these soils which prevent crayfish development [53].

### Synthesis and applications

In spite of the great influence that fishing activities appear to exert on the distribution and activity patterns of Audouin’s gulls, data presented here cannot properly assess the relative importance of natural foraging relative to scavenging on fishing vessels. Fisheries might provide an easyly available food resource, but on the other hand the overexploitation of small pelagic fish stocks, as well as their pelagic predators such as tunas (that during their foraging maneuvers force small pelagic fish to the surface), reduce the availability of natural marine prey to non-diving seabirds, such as Audouin's Gull [13]. This situation implies that local seabird populations might be artificially mantained with discards nowadays, with the supplement of crayfish, while natural fishing at sea could be far more costly. This fact can be a problem, because several studies suggest that the consumption of discards is energetically worse than that of pelagic fish [74, 10]. Nevertheless, we observed that when there are not availability of discards Audouin's Gull also feeds at sea to some extent, both during the day and at night, most probably feeding small pelagic fish.

Fishing pressure has led to widespread overexploitation of fish stocks worldwide, with major impact in long exploited areas such as Europe [75]. Added to the genuine removal of commercial fish, several fleets (particularly trawlers) also produce large amounts of discards, adding unnecessary pressure to the marine environment [76, 77]. Reducing discards has become a priority to help reducing fishing pressure over the oceans, and a first step towards a model of economically and environmentally sustainable fisheries [78, 79]. Within this context, the European Commission took in 2013 the resolution of banning discards in European waters, as part of the last reform of the Common Fisheries Policy [80]. Nevertheless this new scenario does not seem the most optimal for Audouin’s gull, given the great use that this species make of trawling discards. Several studies have shown how the lack of discards produced by trawling moratoria have a negative effect on the breeding performance of this species [81, 82, 83, 84, 12]. The new European scenario could have severe effects on the breeding population of Audouin's gull, as the availability of food at sea might not be enough to sustain the current population, and the American crayfish seems a lower quality prey compared to marine fish [85]. On the other hand, the ban or the reduction of discards would also affect other species of seabirds in the region, and this might increase competition and predation, posing an added potential threat for species of conservation concern [54, 12, 86]. Therefore, the ban on discards should be closely monitored, and efforts should be directed at finding mitigation measures to minimize the impact on the seabird community. Ideally, discard reduction should be based on increased selectivity, and accompanied of improved management of the local fisheries to ensure the recovery of the exploited fish stocks. This would help to gradually reach a natural balance in the populations of predators and preys, which could likely result in a reduction of the population size of some species, including Audouin's Gull [84].

## Supporting Information

S1 Fig Foraging trips of tagged Audouin’s gulls. Orange lines represent the foraging trips. The red circle shows the Audouin’s gull breeding colony (Punta de la Banya, Ebro Delta), where the birds were trapped and GPS-tagged. The modelling range is shown by a black line. The most important fishing ports are also shown (white circles). The area between lines A and B corresponds to the trawling moratorium area.
(DOCX)

S2 Fig Available data for each Audouin’s gull tagged. For each bird, the horizontal (grey) bar shows the period when the GPS provided information. Weekends are highlighted in light grey (vertical bars).
(DOCX)

S3 Fig Environmental variables. Maps for all the environmental variables used in the models are provided (see material and methods for more details).
(DOCX)

S4 Fig Foraging trips duration (h). The median (line), interquartile ranges (box) and minimum and maximum values (dashed lines) are shown. Circles show outlier values.
(DOCX)

S5 Fig Habitat distribution models. Models are presented separately for workdays and weekends, as well as for each time interval.
(DOCX)

S1 Table Tagged Audouin’s gulls information. Detailed information of the Audouin’s gulls (*Larus audouinii*) that provided GPS data: ring; total tracking time (in days); number of locations (both total and only foraging trip-FT- locations, the latter on time also presented separately for weekdays-WD- and weekends-WE), number and duration (in hours) of foraging trips (FT), and number of days for which data were available for modelling.
(DOCX)

S2 Table GPS data available for modelling. For each time interval, the number of birds with at least one location is presented, also considering the number of days available. The gray shadow shows the number of days selected to calibrate the models for each time interval. Information for weekends and workdays is presented separately.
(DOCX)

S3 Table Moonlight information during the study period. Percentage of lunar disk illuminated, and timing of moonrise, moonset, sunrise and sunset. Weekends are showed in light gray. Data from TPE. The Photographer’s Ephemeris. 2013. Software available http://photoephemeris.com/
(DOCX)

S4 Table Spearman correlation matrix. Spearman correlation matrix values for variable selection. In red are shown correlation coefficient values ǀr_s_ǀ ≥0.70. *Variables eliminated prior to the modelling process.
(DOCX)

S1 Text Habitat use cover, Chl-a, and SST calculation. (DOCX)

S2 Text Environmental variables selection procedure. (DOCX)
