# Correction: Modelling Terrestrial and Marine Foraging Habitats in Breeding Audouin's Gulls *Larus audouinii*: Timing Matters

**DOI:** 10.1371/journal.pone.0129989

**Published:** 2015-06-03

**Authors:** Juan Bécares, Manuel García-Tarrasón, Dani Villero, Santiago Bateman, Lluís Jover, Víctor García-Matarranz, Carolina Sanpera, José Manuel Arcos

There is an error in the caption for Fig 3, “AUCs values for workday SDMs resulting from the validation of the models with the sample reserved for validation.” Please see the complete, correct Fig 3 caption here.

**Fig 3:**
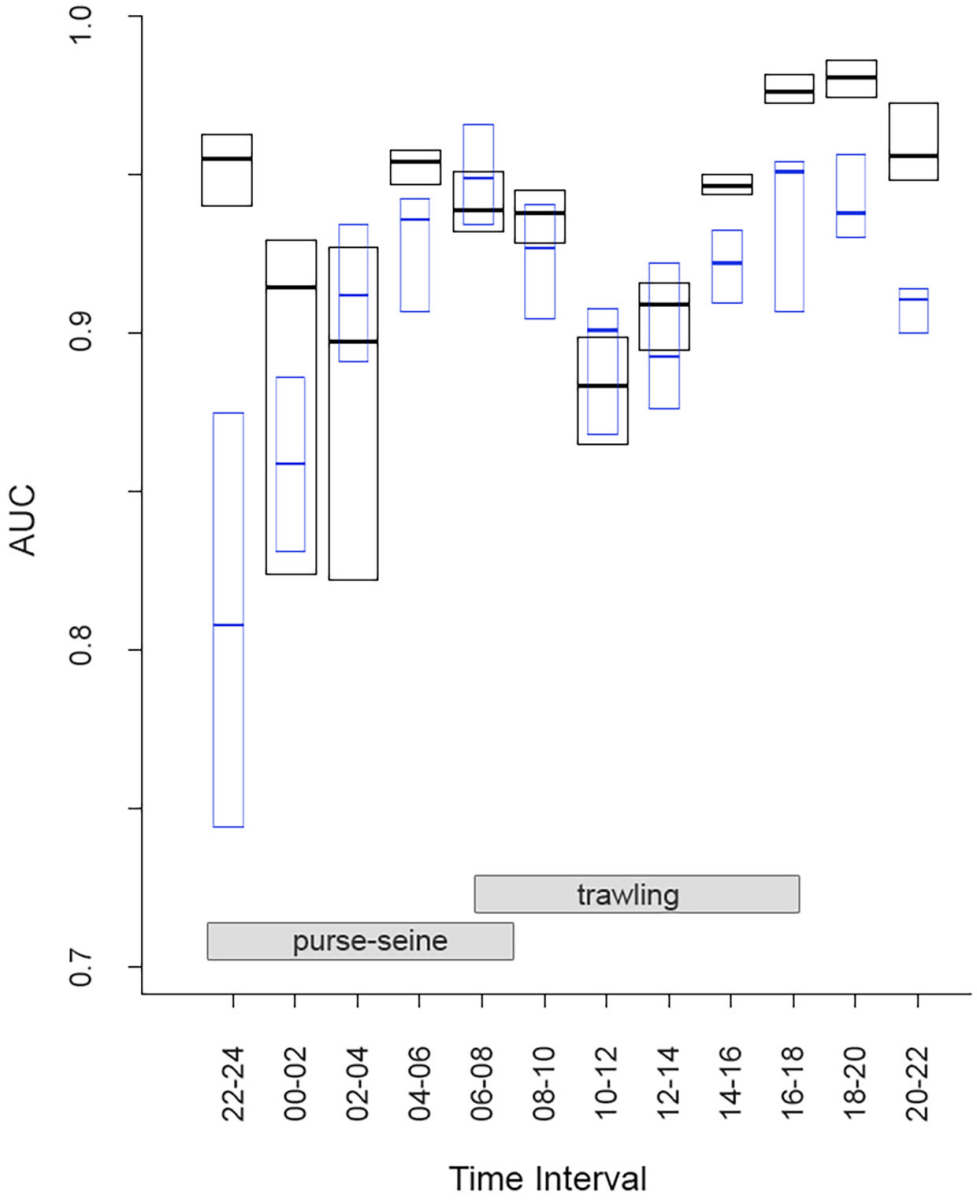
Some examples of Audouin’s gull distribution models for both workdays and weekends. a) purse-seine activity (TI: 02–04h), b) trawling activity (TI:12–14h; A and B lines delimited the area under trawler moratorium) and c) no fishing activity (TI: 18–20h; a coarser scale was selected here, as gull distribution at sea was marginal at this time interval, and focusing on the Ebro Delta allowed to better show the differences between working days and weekends).

There is an error in the caption for Fig 4, “Some examples of Audouin’s gull distribution models for both workdays and weekends.” Please see the complete, correct Fig 4 caption here.

**Fig 4:**
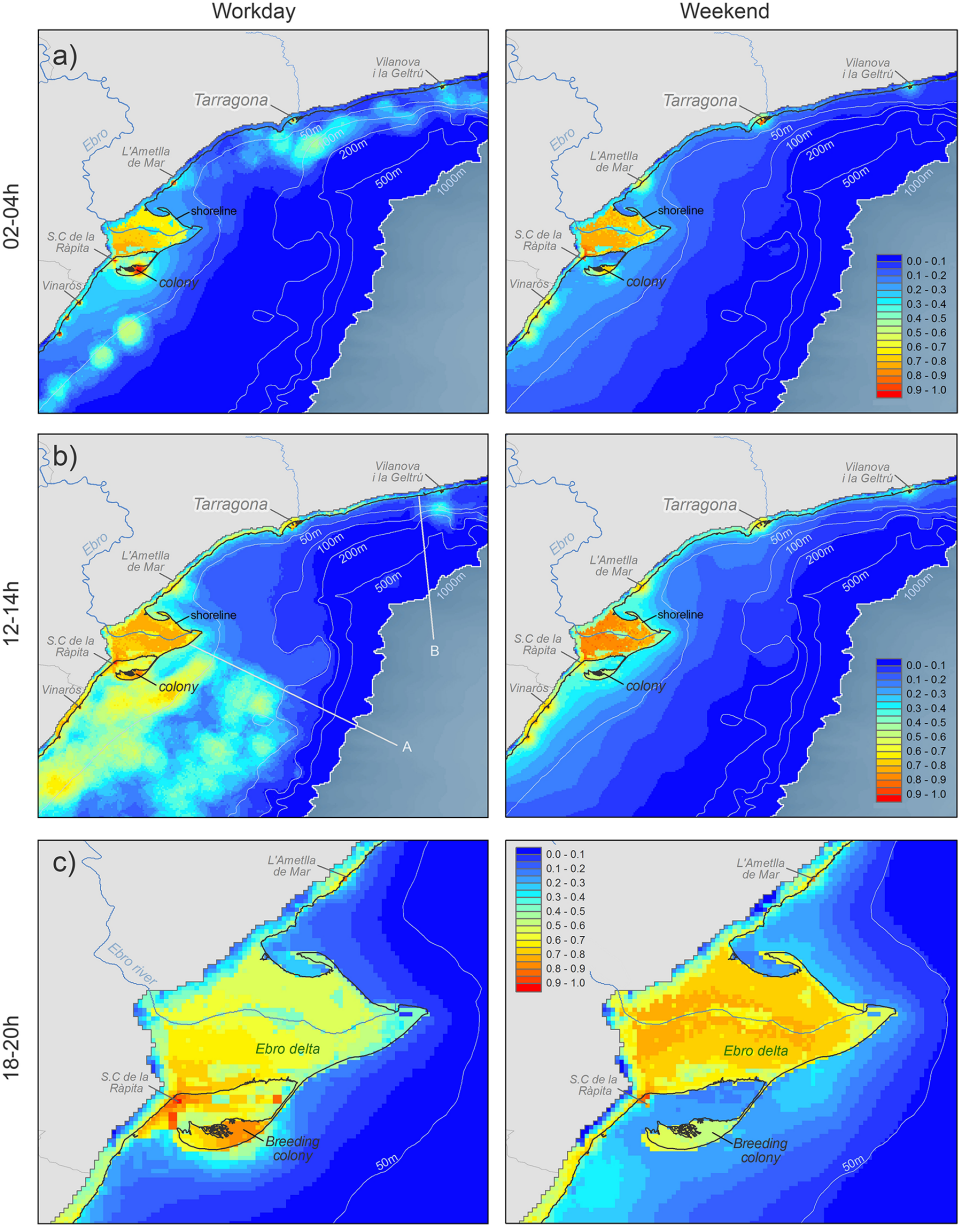
AUCs values for workday SDMs resulting from the validation of the models with the sample reserved for validation. Median (lines) and interquartile ranges (box plots) are shown. Blue boxes show the AUC for cross-validation (cvAUC) between workdays and weekends for each time interval.
