# Supplementary material for: Modelling Terrestrial and Marine Foraging Habitats in Breeding Audouin's Gulls Larus audouinii: Timing Matters
Source: PLoS One. 2015 Apr 14;10(4):e0120799. doi: 10.1371/journal.pone.0120799 (PMC4397092; doi:10.1371/journal.pone.0120799)

**S1** **Fig. Foraging trips** **of tagged Audouin’s gulls.** Orange lines represent the foraging trips. The red circle shows the Audouin’s gull breeding colony (Punta de la Banya, Ebro Delta), where the birds were trapped and GPS-tagged. The modelling range is shown by a black line. The most important fishing ports are also shown (white circles). The area between lines A and B defined the trawling moratorium area.


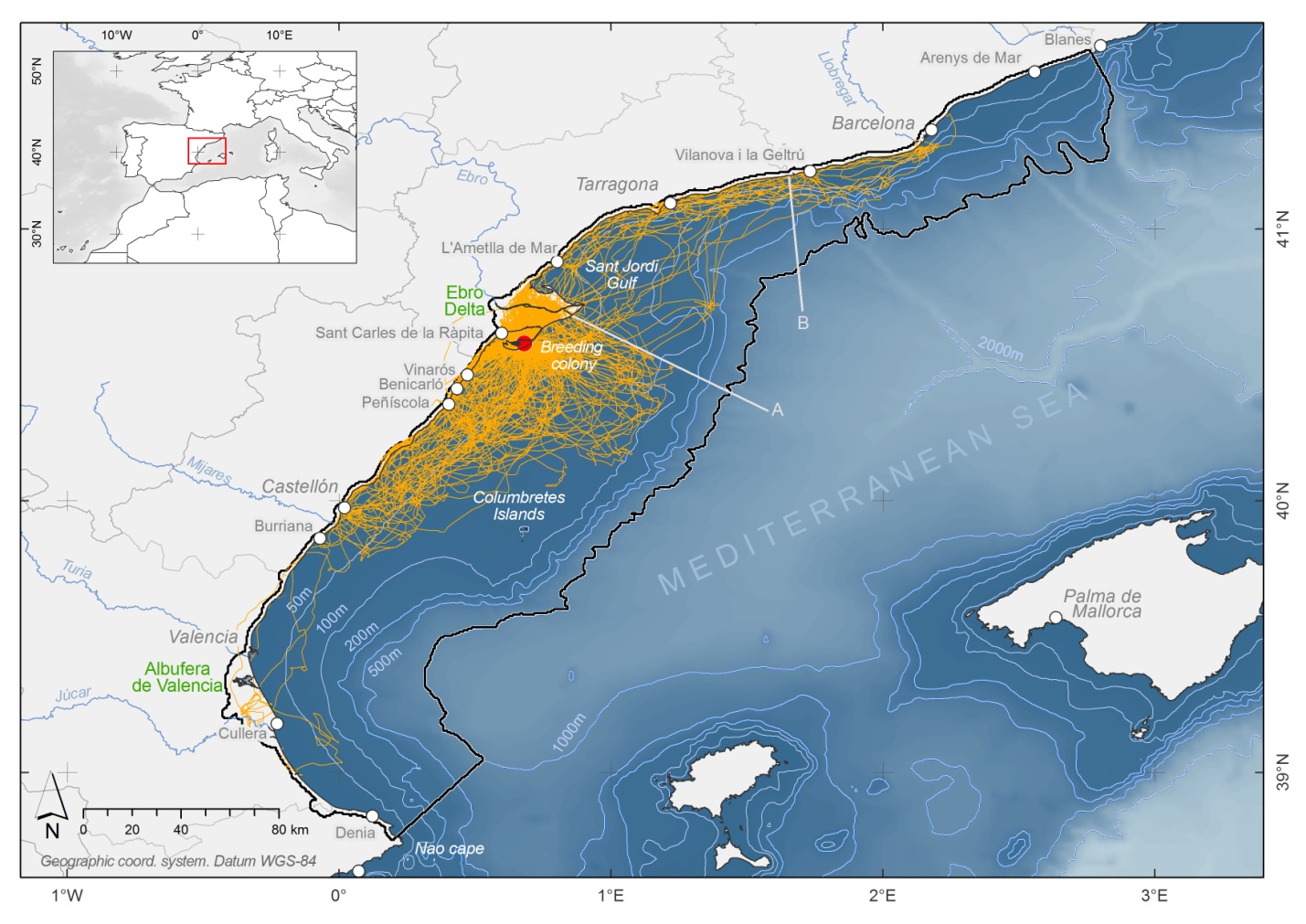

Supplement: S1 Fig — Orange lines represent the foraging trips. The red circle shows the Audouin’s gull breeding colony (Punta de la Banya, Ebro Delta), where the birds were trapped and GPS-tagged. The modelling range is shown by a black line. The most important fishing ports are also shown (white circles). The area between lines A and B corresponds to the trawling moratorium area. (DOCX) [file pone.0120799.s001.docx]
