# Supplementary material for: Modelling Terrestrial and Marine Foraging Habitats in Breeding Audouin's Gulls Larus audouinii: Timing Matters
Source: PLoS One. 2015 Apr 14;10(4):e0120799. doi: 10.1371/journal.pone.0120799 (PMC4397092; doi:10.1371/journal.pone.0120799)

**S2 Fig.** **Available data for each Audouin’s gull tagged.** For each bird, the horizontal (grey) bar shows the period when the GPS provided information. Weekends are highlighted in light grey (vertical bars).


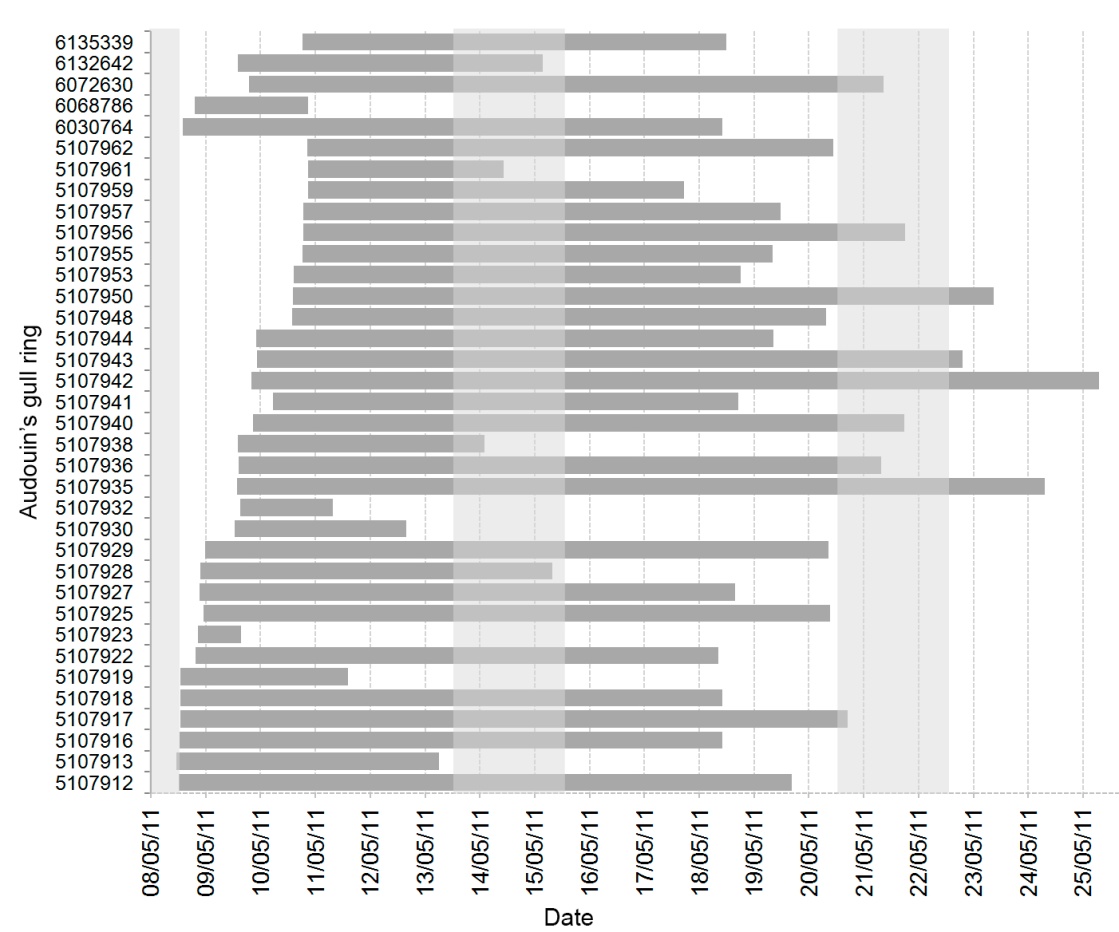

Supplement: S2 Fig — For each bird, the horizontal (grey) bar shows the period when the GPS provided information. Weekends are highlighted in light grey (vertical bars). (DOCX) [file pone.0120799.s002.docx]
