# Supplementary material for: Modelling Terrestrial and Marine Foraging Habitats in Breeding Audouin's Gulls Larus audouinii: Timing Matters
Source: PLoS One. 2015 Apr 14;10(4):e0120799. doi: 10.1371/journal.pone.0120799 (PMC4397092; doi:10.1371/journal.pone.0120799)

**S3 Fig. Environmental variables.** Maps for all the environmental variables used in the models are provided (see material and methods for more details).


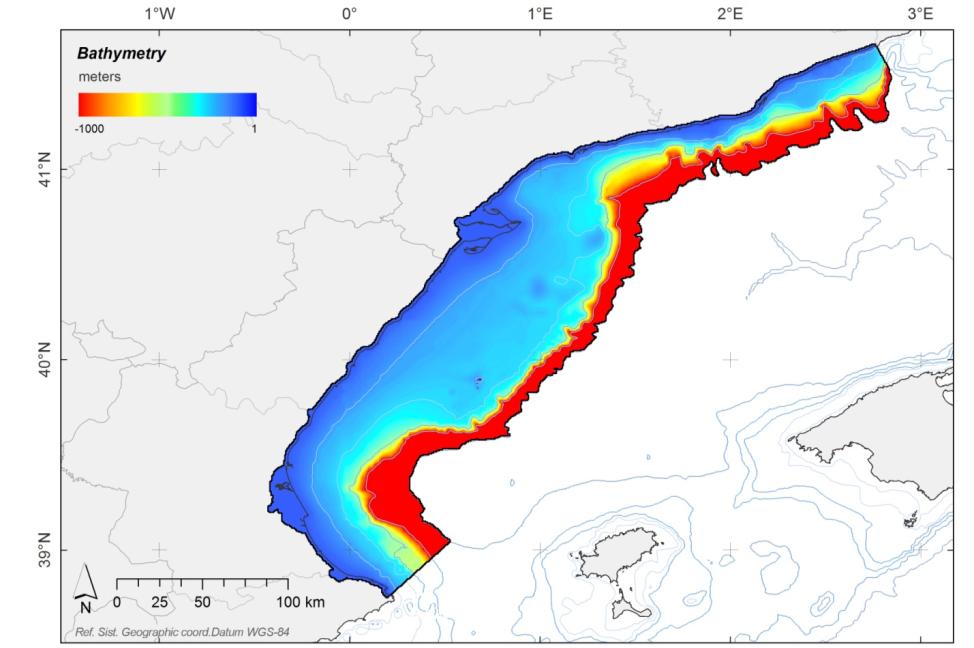


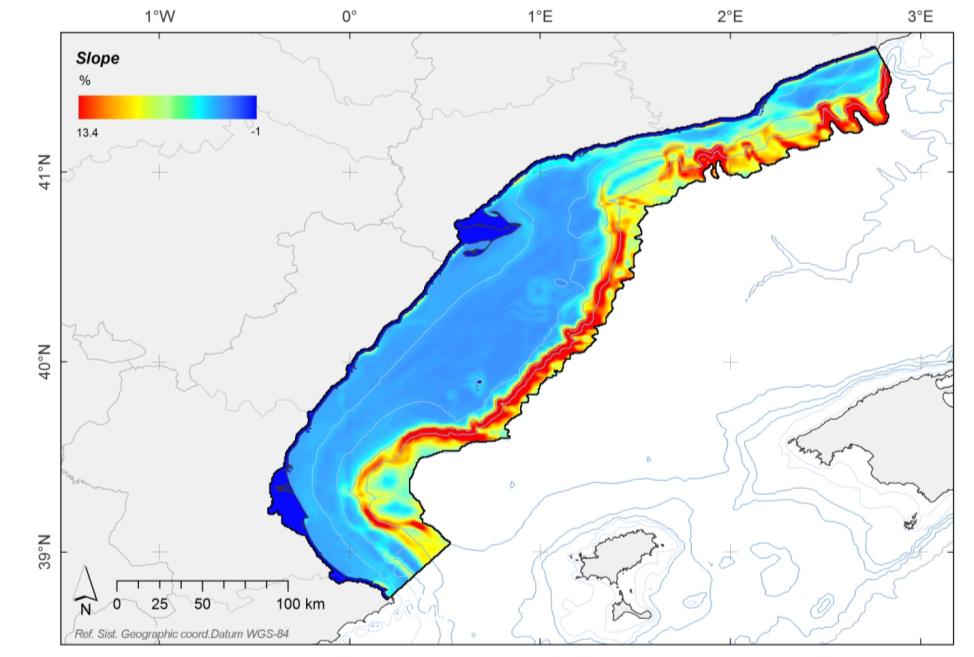


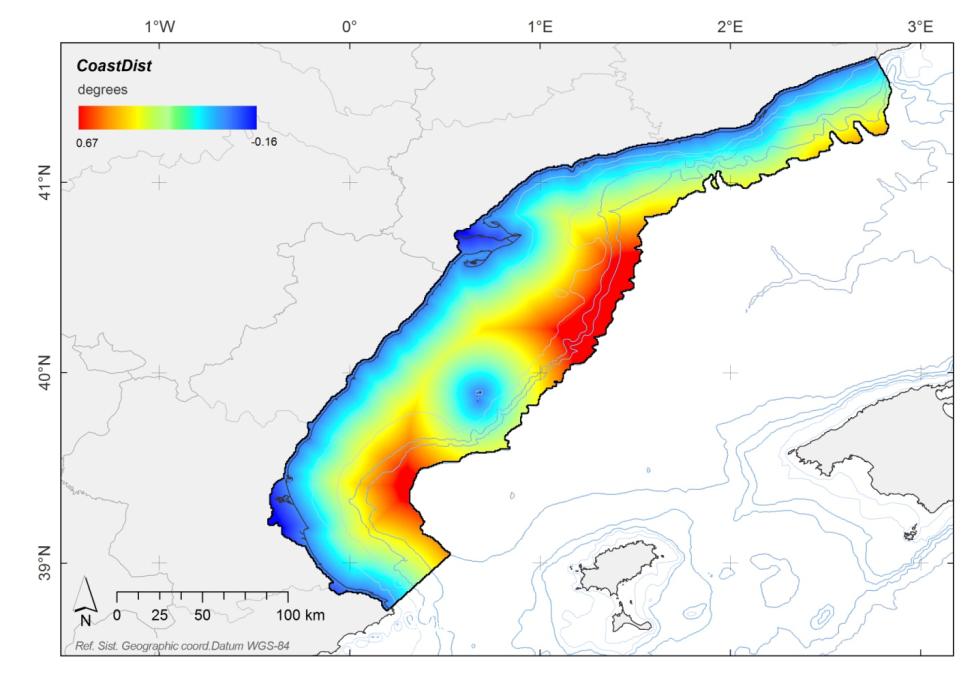


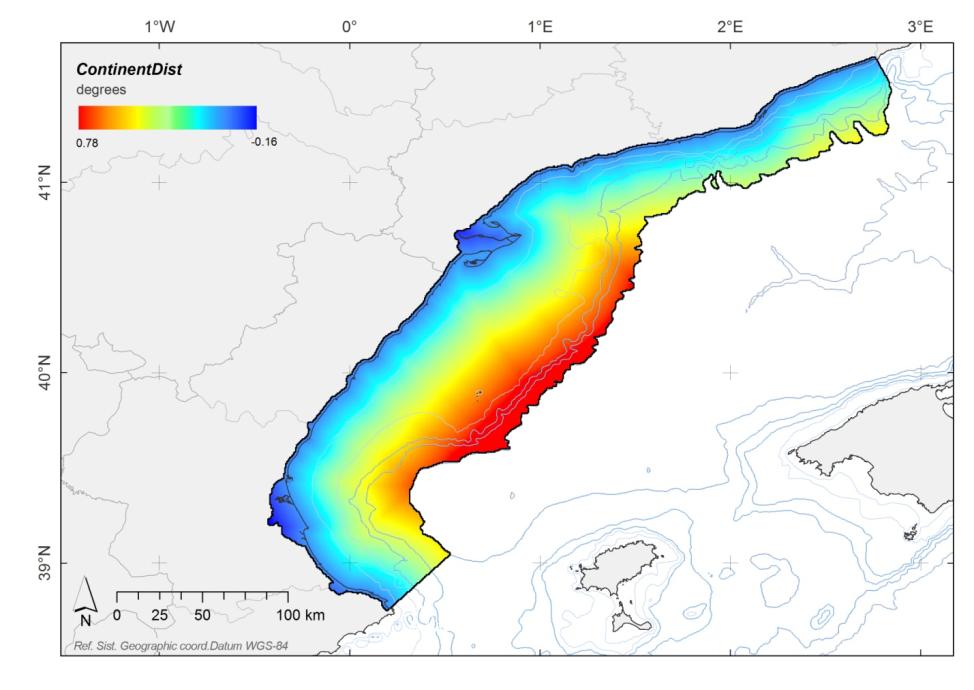


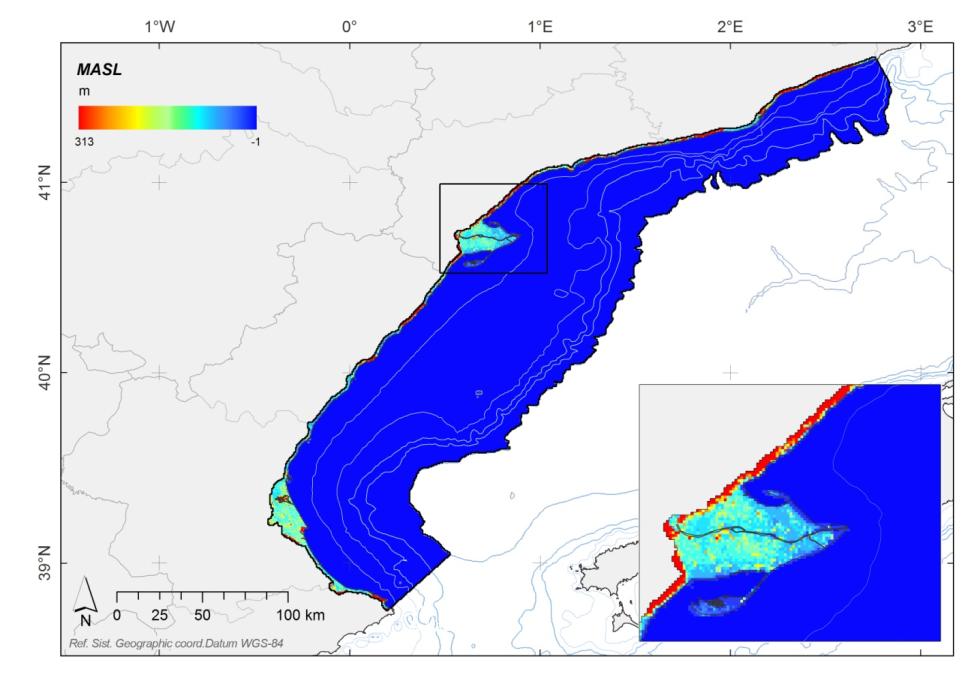


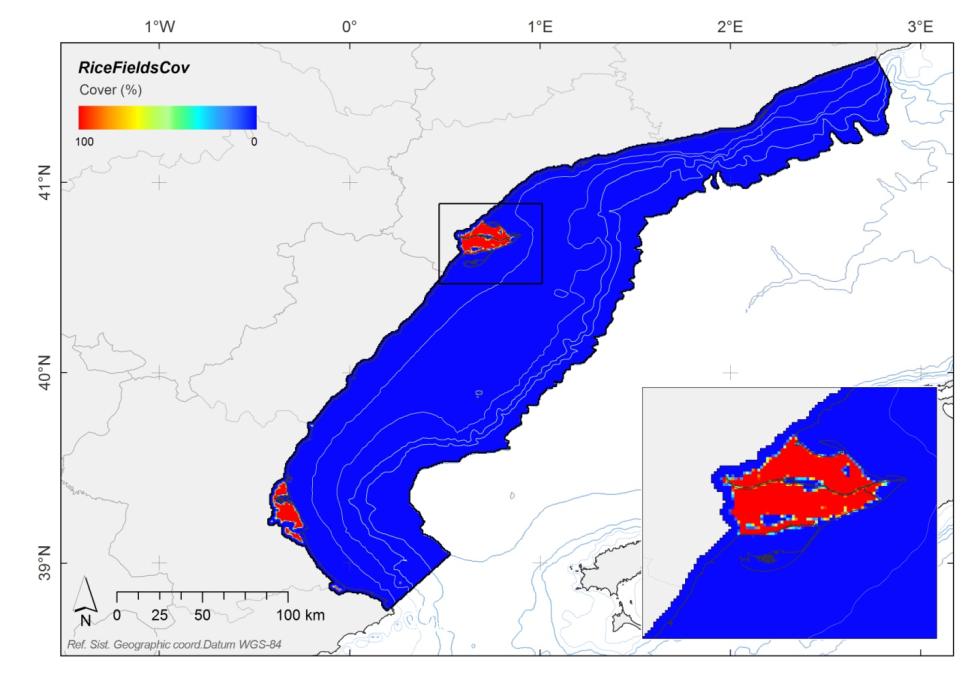


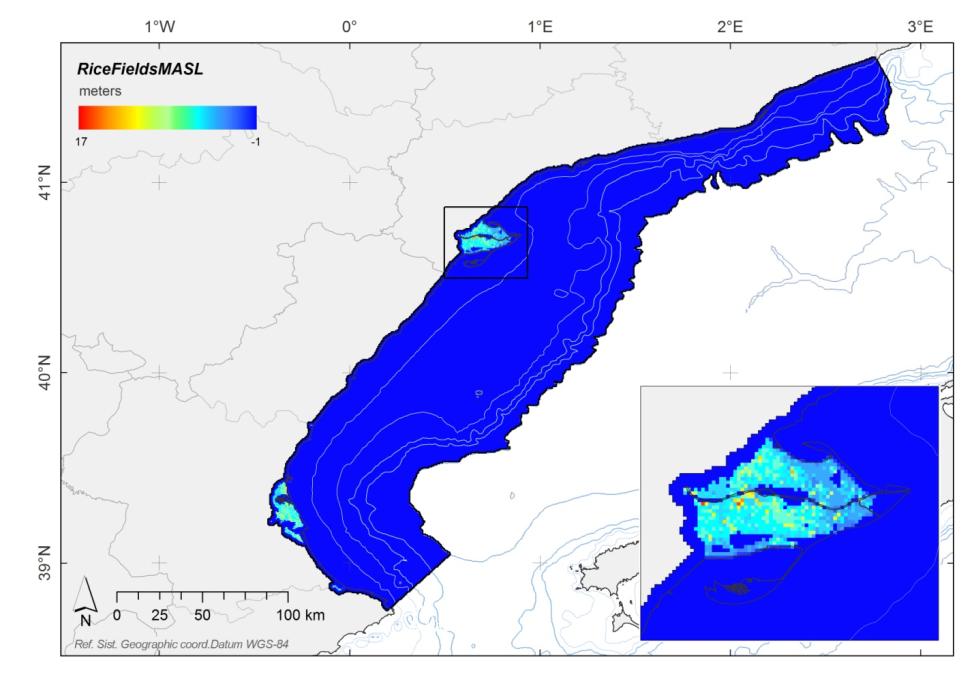


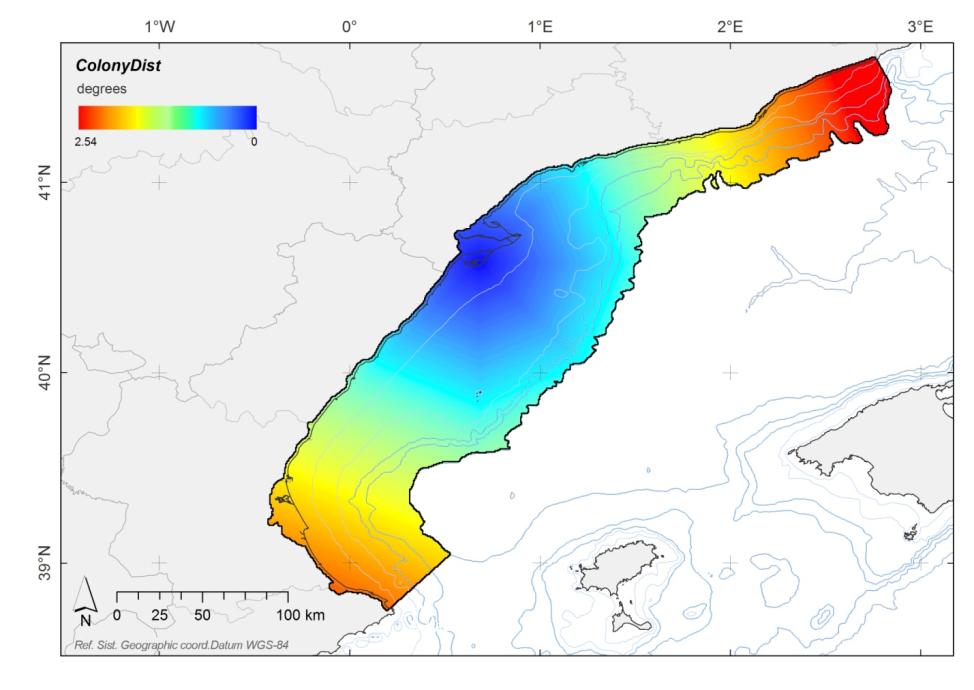


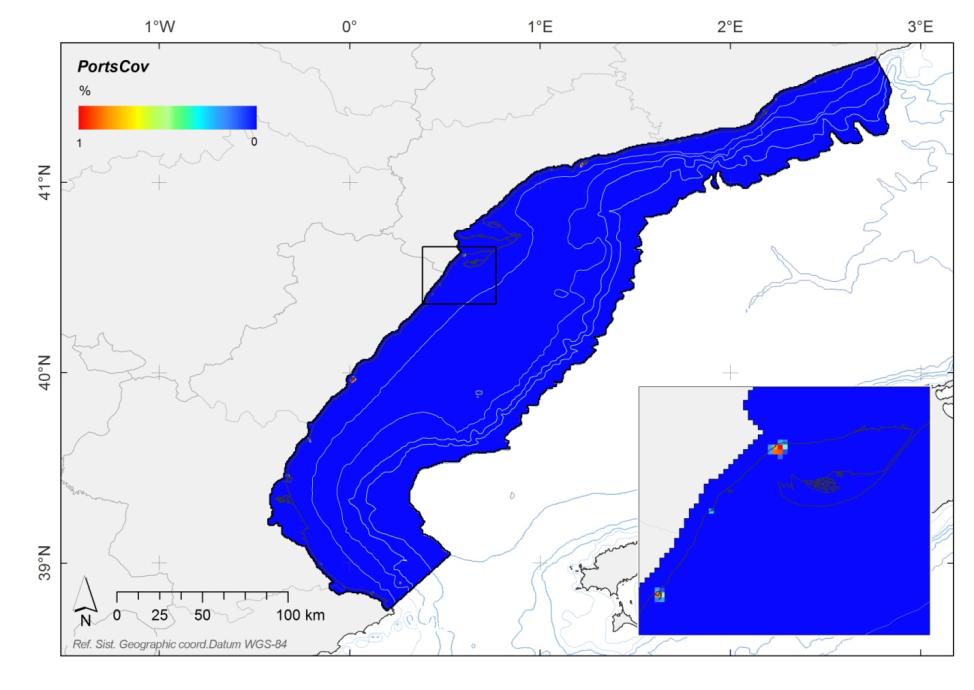

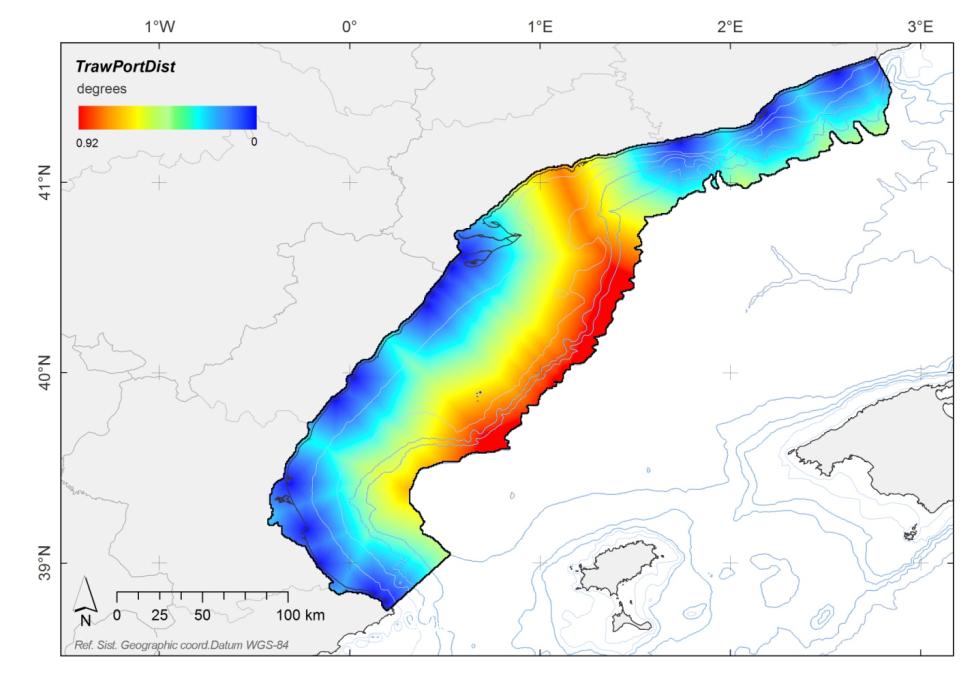


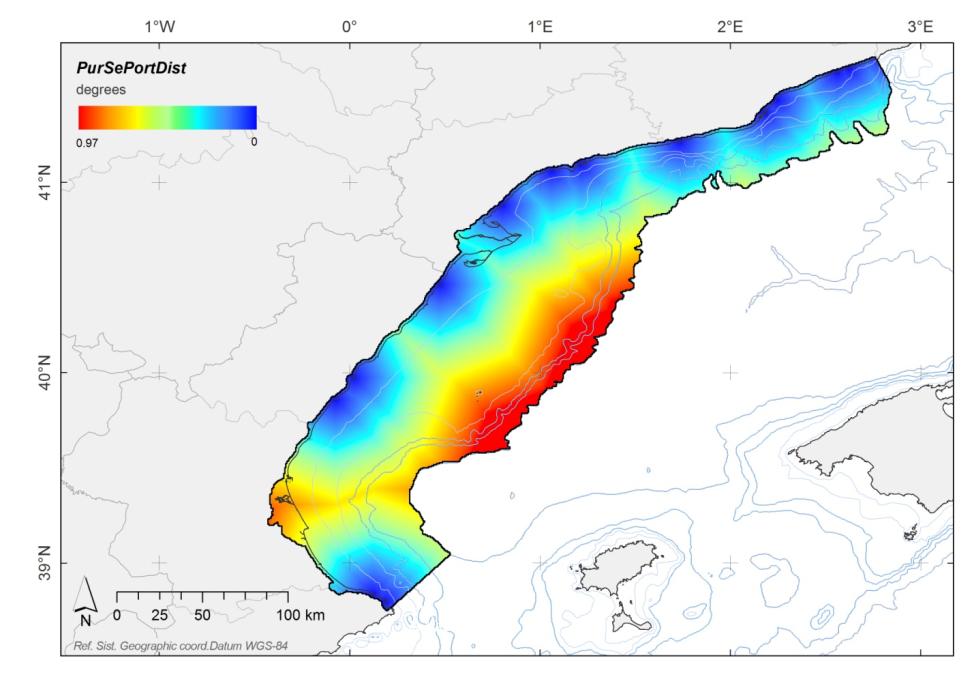


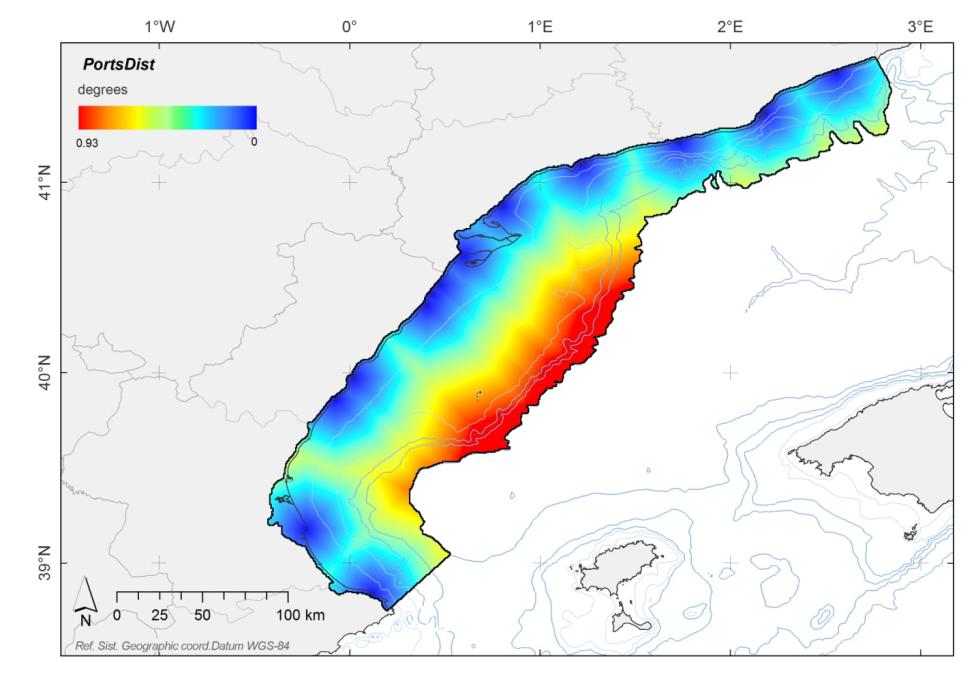


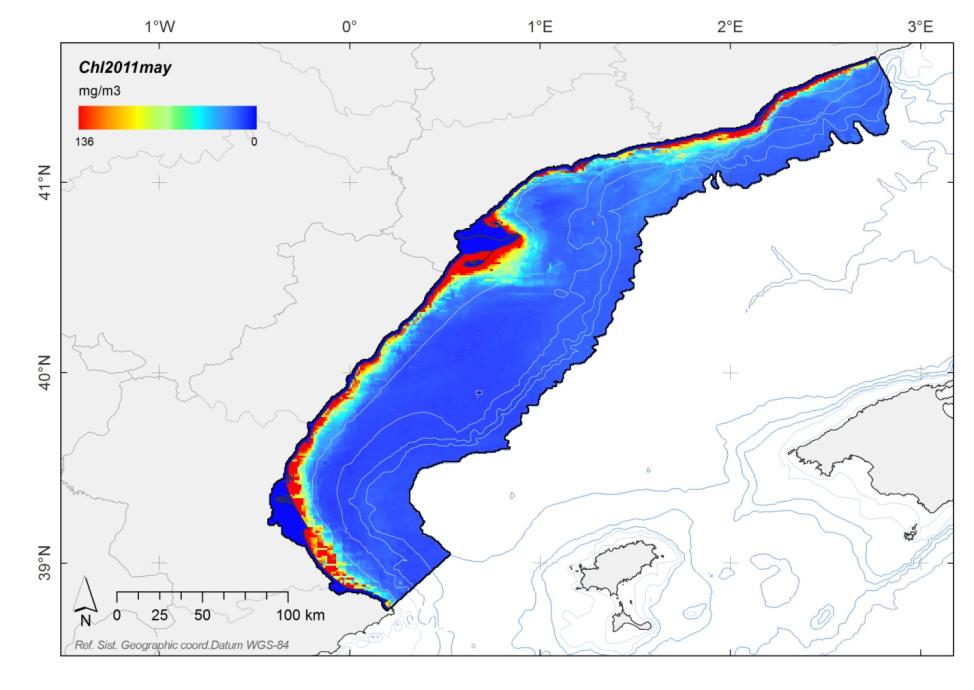


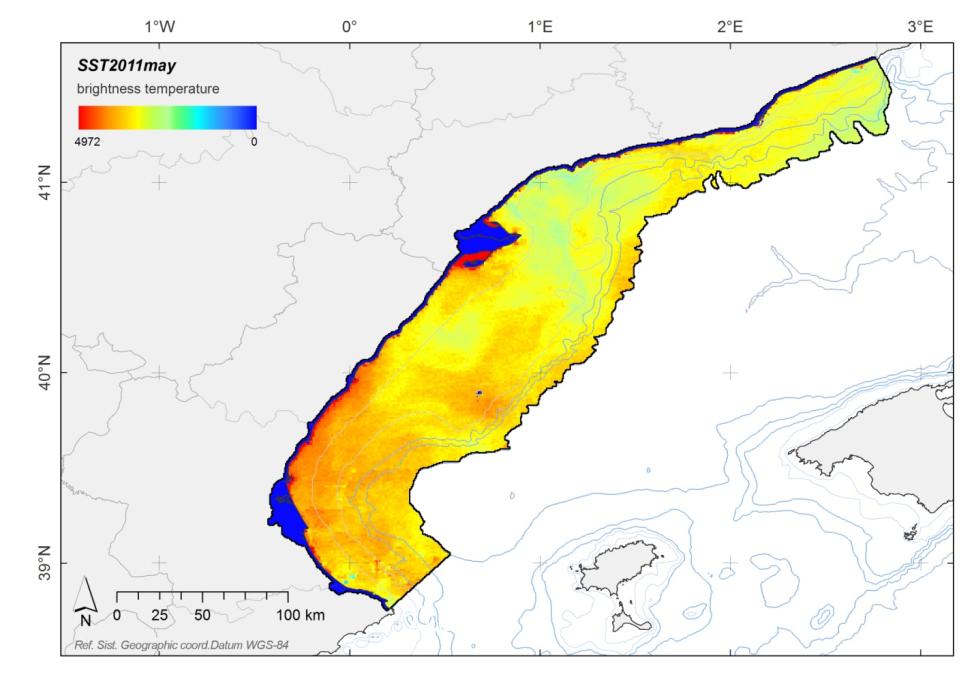


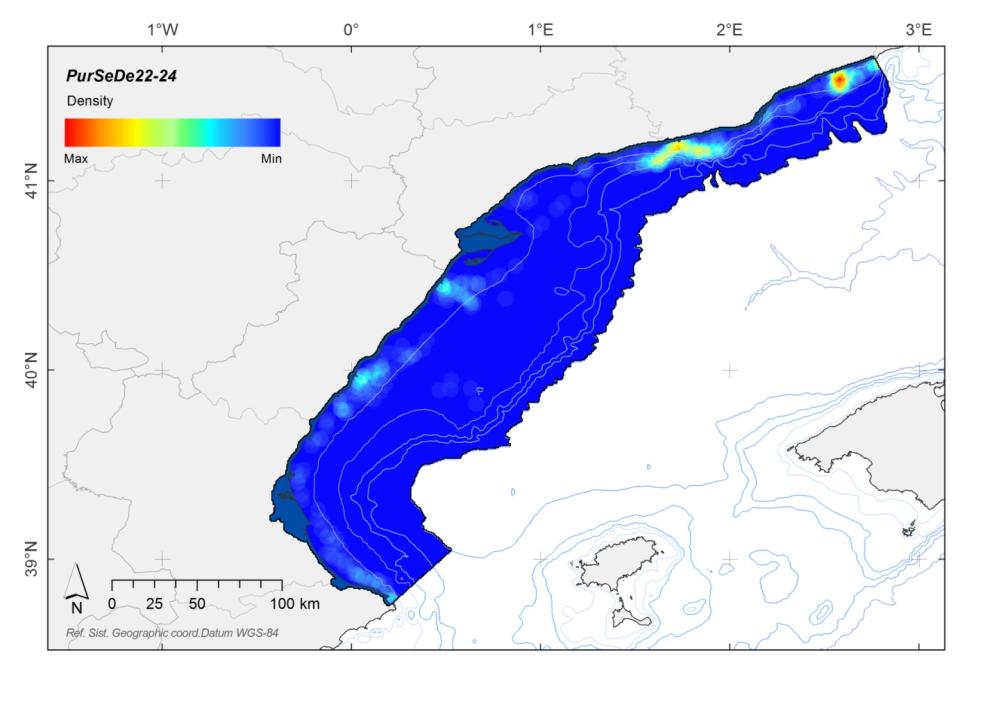

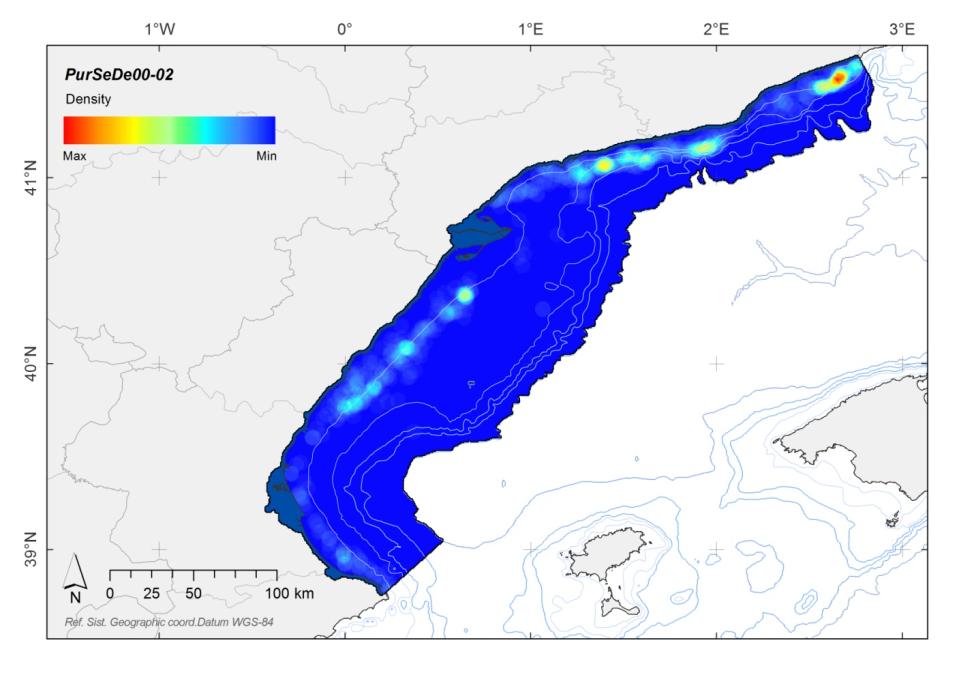


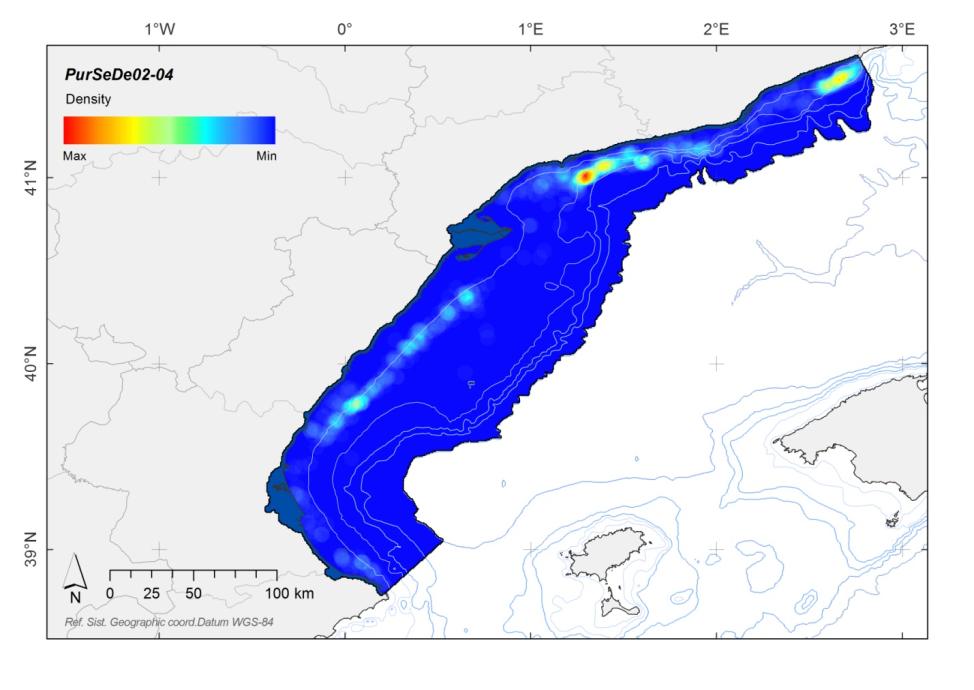


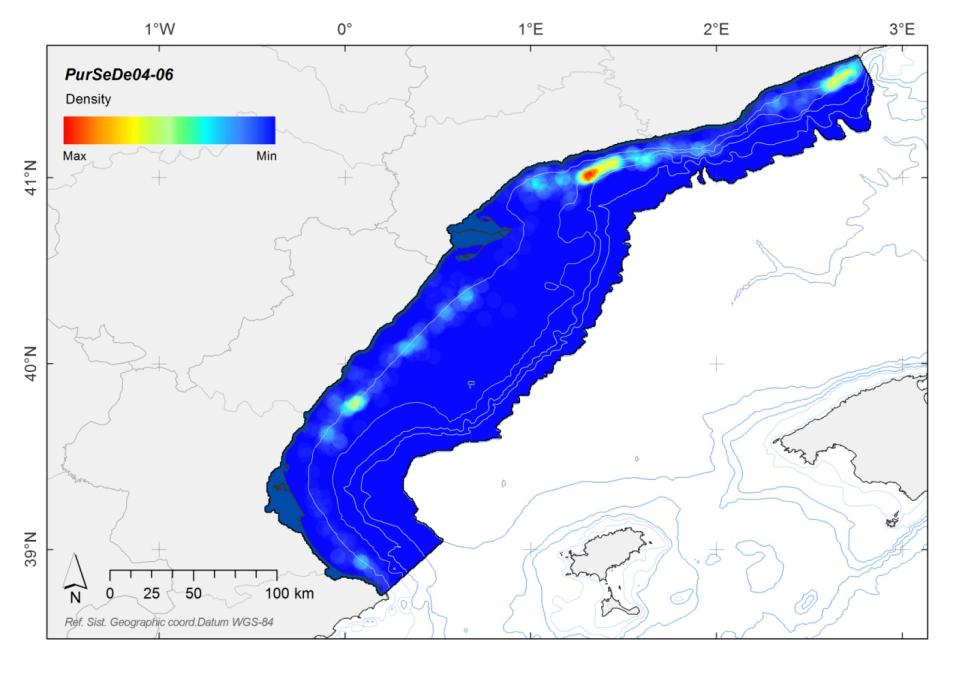


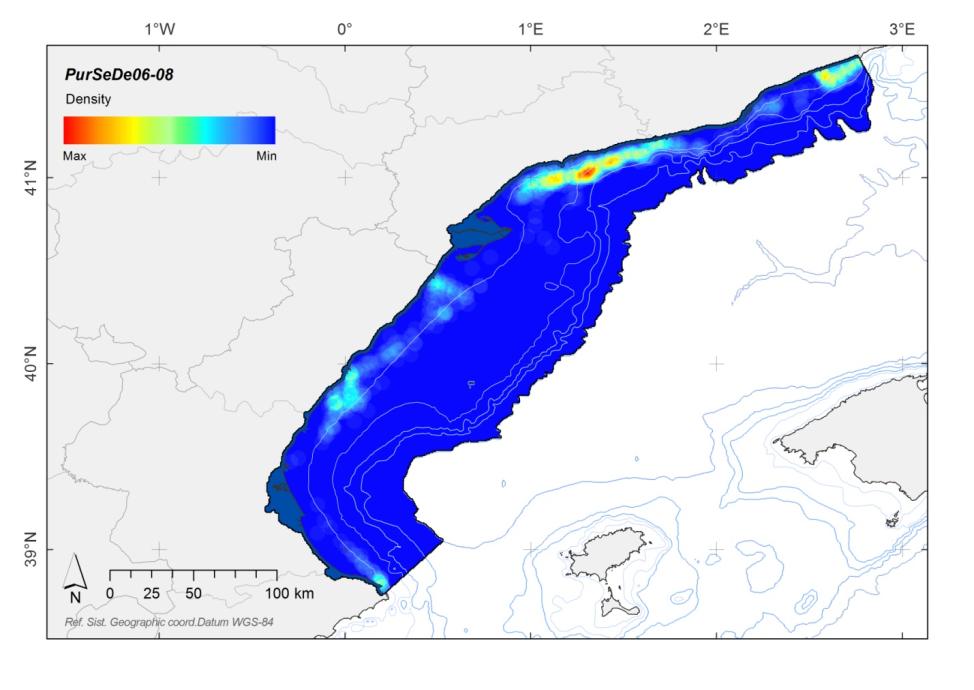


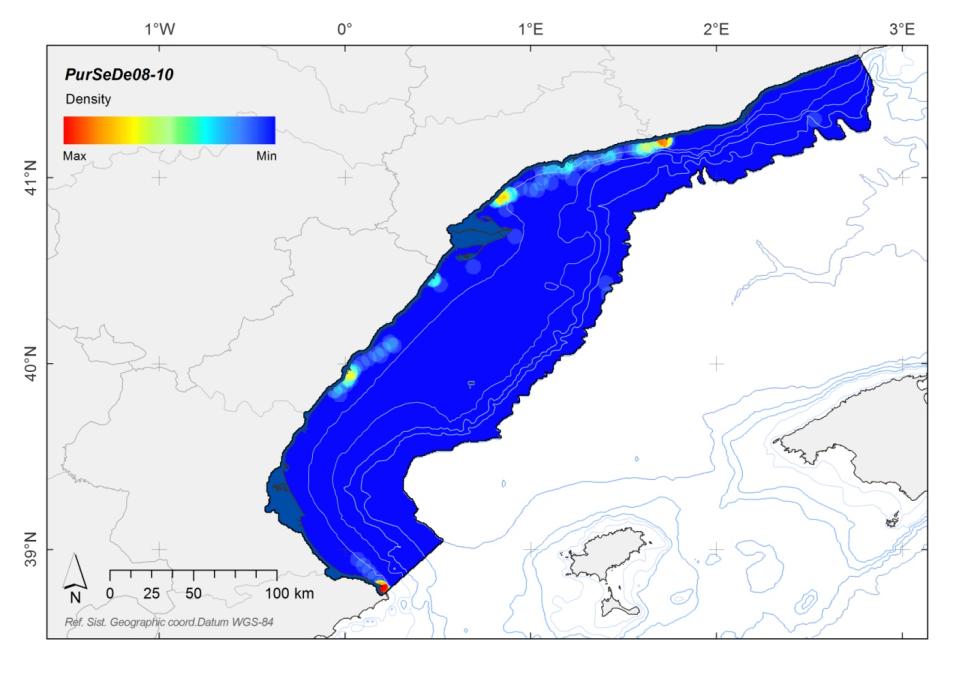


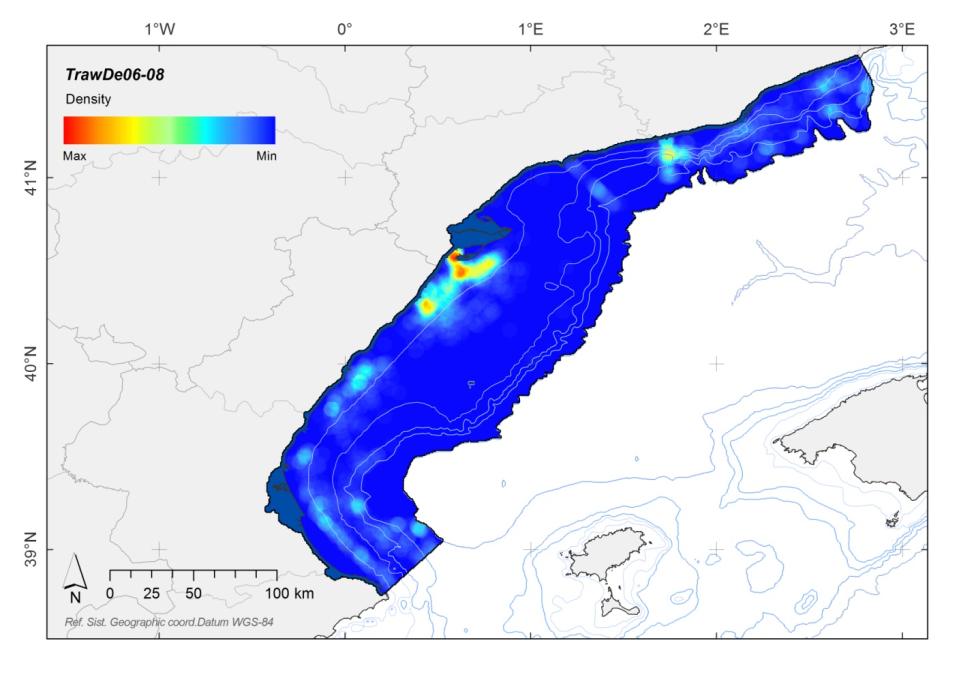


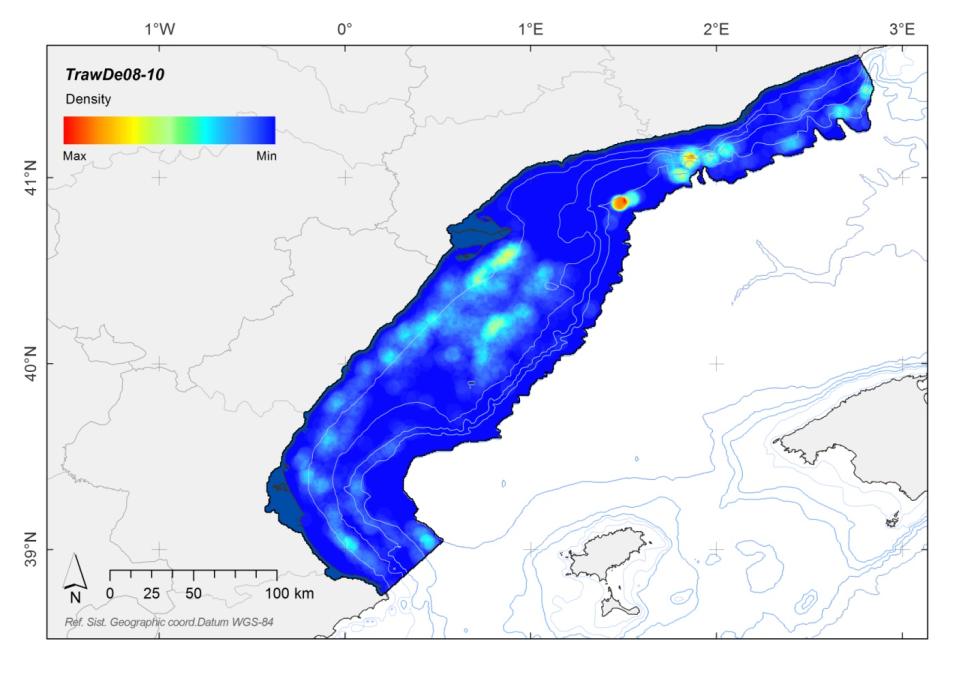


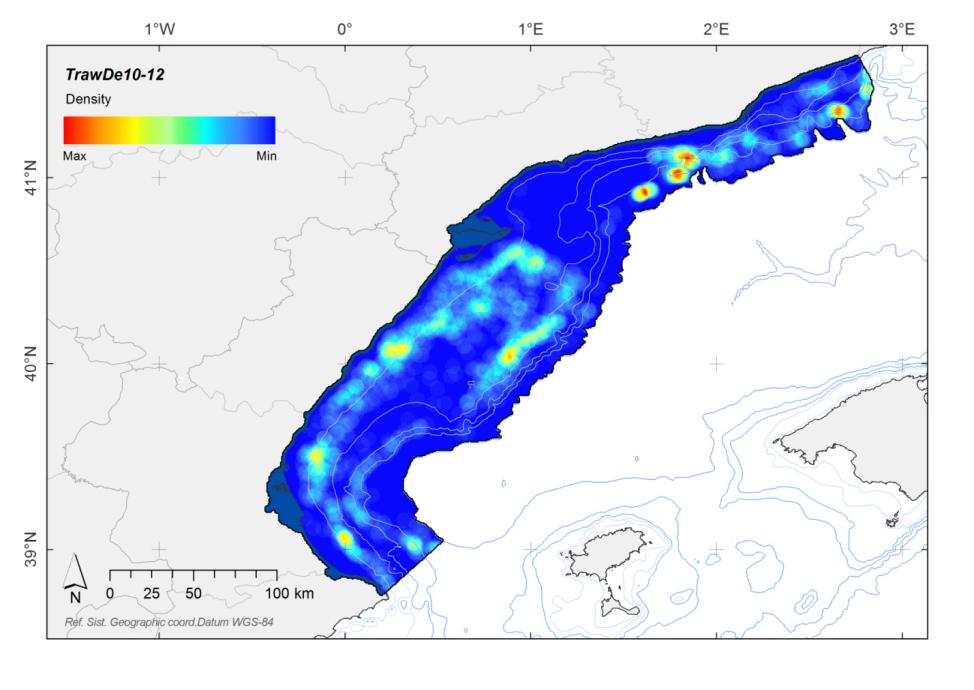


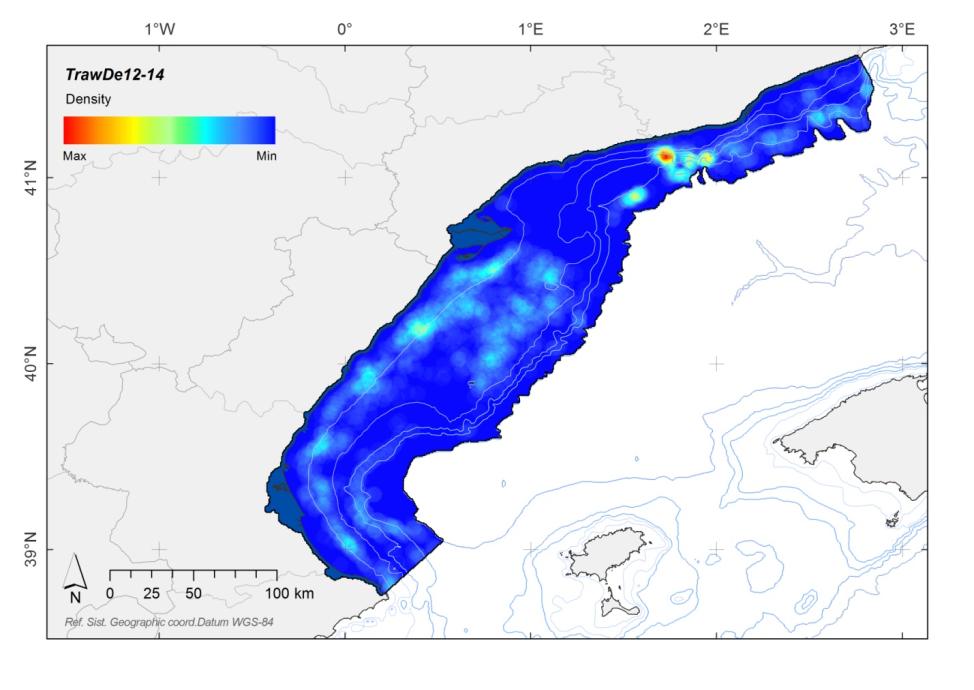


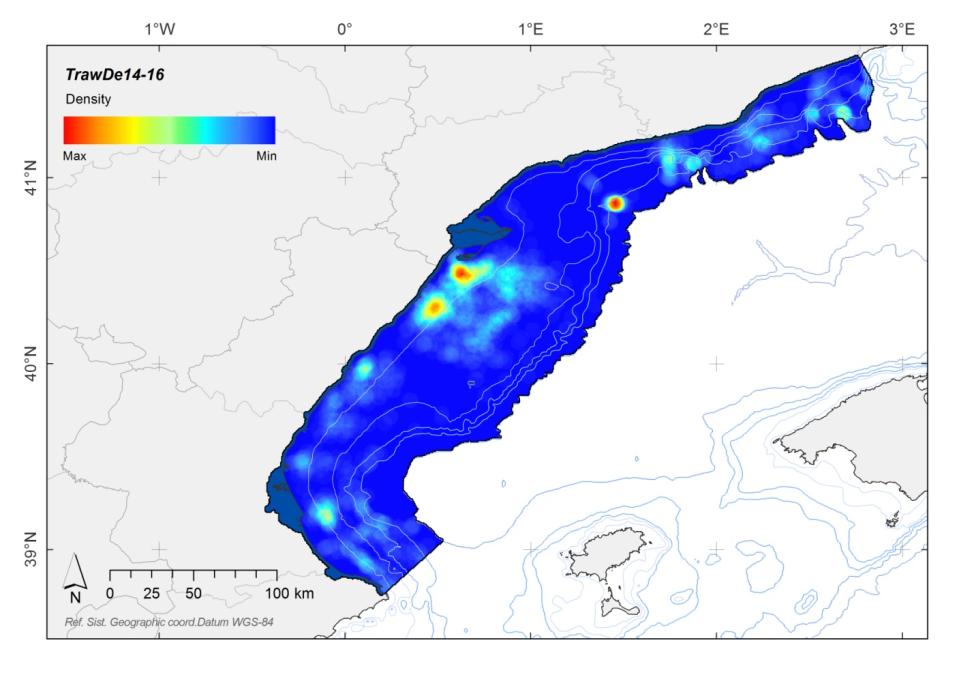


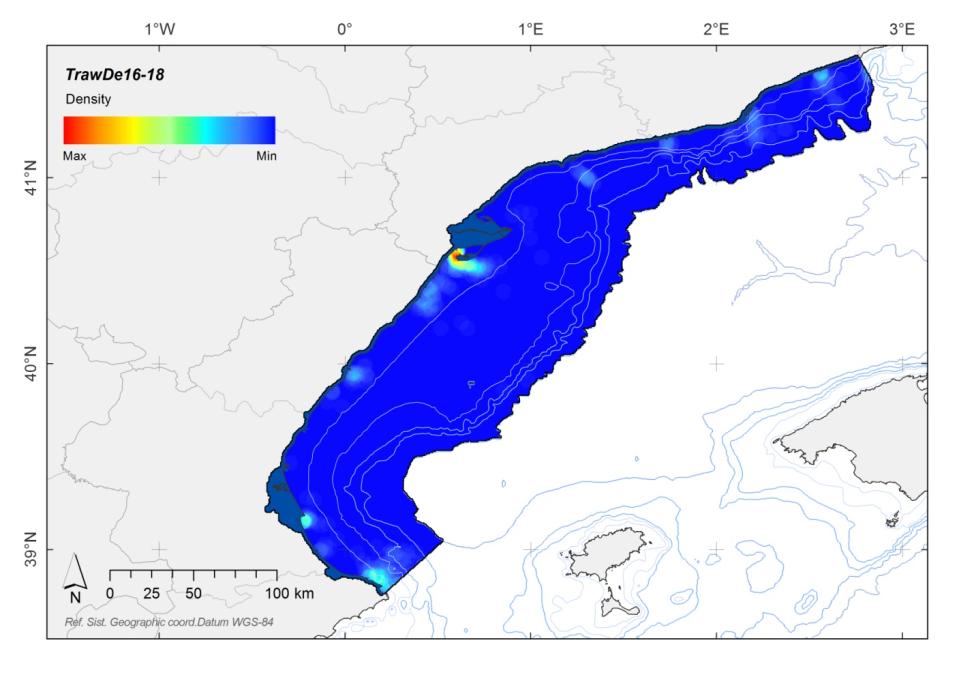

Supplement: S3 Fig — Maps for all the environmental variables used in the models are provided (see material and methods for more details). (DOCX) [file pone.0120799.s003.docx]
