# Supplementary material for: Modelling Terrestrial and Marine Foraging Habitats in Breeding Audouin's Gulls Larus audouinii: Timing Matters
Source: PLoS One. 2015 Apr 14;10(4):e0120799. doi: 10.1371/journal.pone.0120799 (PMC4397092; doi:10.1371/journal.pone.0120799)

**S4 Fig.** **Foraging trips Duration (h).** The median (line), interquartile ranges (box) and minimum and maximum values (dashed lines) are shown. Circles show outlier values.


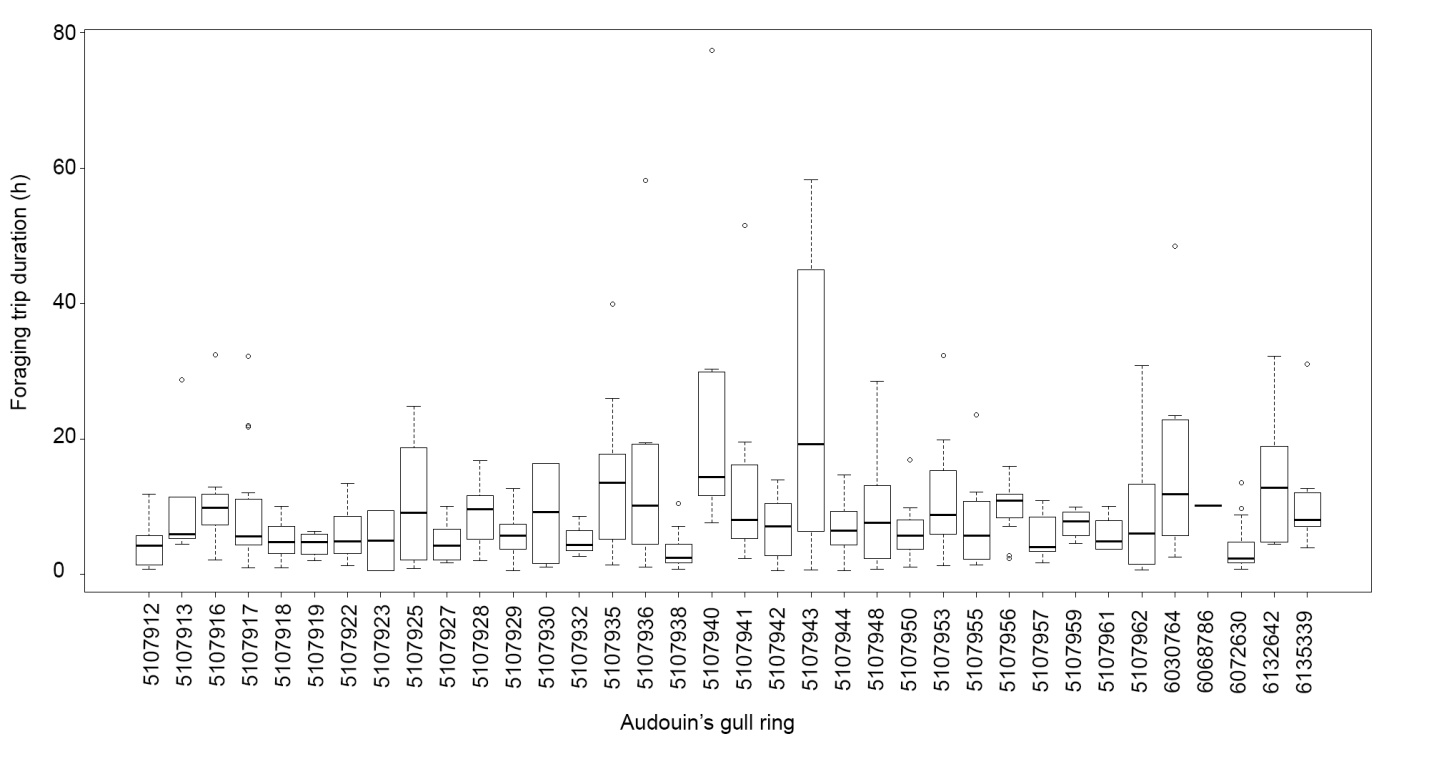

Supplement: S4 Fig — The median (line), interquartile ranges (box) and minimum and maximum values (dashed lines) are shown. Circles show outlier values. (DOCX) [file pone.0120799.s004.docx]
