# Supplementary material for: Modelling Terrestrial and Marine Foraging Habitats in Breeding Audouin's Gulls Larus audouinii: Timing Matters
Source: PLoS One. 2015 Apr 14;10(4):e0120799. doi: 10.1371/journal.pone.0120799 (PMC4397092; doi:10.1371/journal.pone.0120799)
Supplement: S1 Table — Detailed information of the Audouin’s gulls (Larus audouinii) that provided GPS data: ring; total tracking time (in days); number of locations (both total and only foraging trip-FT- locations, the latter on time also presented separately for weekdays-WD- and weekends-WE), number and duration (in hours) of foraging trips (FT), and number of days for which data were available for modelling. (DOCX) [file pone.0120799.s006.docx]

**S1 Table. Tagged Audouin’s gulls information.** Detailed information of the Audouin’s gulls (*Larus audouinii*) that provided GPS data: ring; total tracking time (in days); number of locations (both total and only foraging trip -FT- locations, the latter on time also presented separately for weekdays-WD- and weekends -WE), number and duration (in hours) of foraging trips (FT), and number of days for which data were available for modelling.

| Metallic ring | Track time (days) | Nº locations | | | | Nº FT | Average FT time (hours) ± ED | Available days | |
| --- | --- | --- | --- | --- | --- | --- | --- | --- | --- |
|  |  | Total | FT | FT WD | FT WE |  |  | WD | WE |
| **5107912** | 11.23 | 3175 | 915 | 704 | 211 | 17 | 4.5 ± 3.7 | 9 | 3 |
| **5107913** | 4.79 | 1380 | 669 | 514 | 155 | 5 | 11.2 ± 10.2 | 4 | 1 |
| **5107916** | 9.91 | 2834 | 1110 | 687 | 423 | 8 | 11. 6 ± 9.0 | 7 | 2 |
| **5107917** | 12.20 | 3508 | 1981 | 1483 | 498 | 18 | 9.2 ± 8.2 | 10 | 3 |
| **5107918** | 9.89 | 2794 | 747 | 577 | 170 | 12 | 5.2 ± 2.9 | 7 | 3 |
| **5107919** | 3.06 | 875 | 215 | 191 | 24 | 4 | 4.5 ± 1.9 | 3 | 1 |
| **5107922** | 9.55 | 2741 | 1092 | 792 | 300 | 15 | 6.1 ± 4.0 | 8 | 3 |
| **5107923** | 0.82 | 237 | 119 | 113 | 6 | 2 | 5.0 ± 6.3 | 1 | 1 |
| **5107925** | 11.55 | 3300 | 1245 | 1144 | 101 | 10 | 10.4 ± 8.6 | 10 | 2 |
| **5107927** | 9.79 | 2796 | 897 | 706 | 191 | 16 | 4.7 ± 2.7 | 8 | 2 |
| **5107928** | 6.44 | 1826 | 745 | 629 | 116 | 7 | 8.9 ± 5.3 | 5 | 1 |
| **5107929** | 11.48 | 3280 | 803 | 690 | 113 | 12 | 5.6 ± 3.2 | 9 | 2 |
| **5107930** | 3.17 | 911 | 431 | 431 | 0 | 4 | 9.0 ± 8.5 | 4 | 0 |
| **5107932** | 1.69 | 509 | 187 | 187 | 0 | 3 | 5.2 ± 3.1 | 3 | 0 |
| **5107935** | 14.75 | 4222 | 2386 | 1880 | 506 | 15 | 13.3 ± 10.5 | 12 | 4 |
| **5107936** | 11.75 | 3395 | 1807 | 1330 | 477 | 10 | 15.1 ± 16.6 | 10 | 3 |
| **5107938** | 4.49 | 1283 | 410 | 410 | 0 | 9 | 3.8 ± 3.2 | 5 | 0 |
| **5107940** | 11.96 | 3428 | 2189 | 1465 | 724 | 7 | 26.1 ± 24.4 | 9 | 3 |
| **5107941** | 8.92 | 2561 | 1353 | 966 | 387 | 8 | 14.1 ± 16.1 | 7 | 2 |
| **5107942** | 15.50 | 4418 | 1861 | 1378 | 483 | 23 | 6.7 ± 4.2 | 12 | 4 |
| **5107943** | 15.60 | 4467 | 2401 | 1392 | 1009 | 8 | 25.0 ± 22.6 | 9 | 4 |
| **5107944** | 9.53 | 2693 | 879 | 757 | 122 | 11 | 6.7 ± 4.4 | 8 | 2 |
| **5107948** | 9.77 | 2795 | 1283 | 991 | 292 | 12 | 8.9 ± 8.2 | 9 | 2 |
| **5107950** | 12.80 | 3652 | 950 | 598 | 352 | 13 | 6.1 ± 4.2 | 8 | 4 |
| **5107953** | 8.15 | 2324 | 1021 | 798 | 223 | 7 | 12.2 ± 10.7 | 7 | 2 |
| **5107955** | 8.59 | 2461 | 1051 | 721 | 330 | 12 | 7.3 ± 6.4 | 7 | 2 |
| **5107956** | 11.01 | 3140 | 1427 | 1118 | 309 | 12 | 9.9 ± 4.1 | 9 | 2 |
| **5107957** | 8.72 | 2495 | 829 | 605 | 224 | 13 | 5.3 ± 3.2 | 7 | 2 |
| **5107959** | 6.94 | 1986 | 542 | 423 | 119 | 6 | 7.5 ± 2.1 | 5 | 2 |
| **5107961** | 3.63 | 1046 | 421 | 376 | 45 | 6 | 5.9 ± 2.6 | 4 | 1 |
| **5107962** | 9.64 | 2691 | 1215 | 993 | 222 | 11 | 9.2 ± 10.1 | 9 | 2 |
| **6030764** | 9.85 | 2795 | 1580 | 1235 | 345 | 8 | 16.5 ± 15.1 | 7 | 2 |
| **6068786** | 2.08 | 600 | 244 | 244 | 0 | 2 | 10.2 ± 0.1 | 2 | 0 |
| **6072630** | 11.59 | 3339 | 1079 | 839 | 240 | 24 | 3.8 ± 3.2 | 9 | 3 |
| **6132642** | 5.55 | 1615 | 1031 | 736 | 295 | 6 | 14.3 ± 10.4 | 5 | 2 |
| **6135339** | 7.76 | 2228 | 975 | 741 | 234 | 7 | 11.6 ± 9.1 | 6 | 2 |
