# Supplementary material for: Modelling Terrestrial and Marine Foraging Habitats in Breeding Audouin's Gulls Larus audouinii: Timing Matters
Source: PLoS One. 2015 Apr 14;10(4):e0120799. doi: 10.1371/journal.pone.0120799 (PMC4397092; doi:10.1371/journal.pone.0120799)
Supplement: S2 Table — For each time interval, the number of birds with at least one location is presented, also considering the number of days available. The gray shadow shows the number of days selected to calibrate the models for each time interval. Information for weekends and workdays is presented separately. (DOCX) [file pone.0120799.s007.docx]

S2 Table. GPS data available for modelling. For each time interval, the number of birds with at least one location is presented, also considering the number of days available. The gray shadow shows the number of days selected to calibrate the models for each time interval. Information for weekends and workdays is presented separately.

Weekends

| **Available days**  **of data** | **00-02** | **02-04** | **04-06** | **06-08** | **08-10** | **10-12** | **12-14** | **14-16** | **16-18** | **18-20** | **20-22** | **22-24** |
| --- | --- | --- | --- | --- | --- | --- | --- | --- | --- | --- | --- | --- |
| 1 day | 15 | 15 | 18 | 20 | 23 | 24 | 24 | 28 | 27 | 26 | 26 | 24 |
| 2 days | 6 | 5 | 7 | 11 | 10 | 11 | 14 | 15 | 15 | 14 | 14 | 8 |
| 3 days | 2 | 2 | 4 | 5 | 3 | 3 | 2 | 1 | 3 | 5 | 4 | 2 |
| 4 days | 0 | 0 | 0 | 1 | 2 | 1 | 1 | 1 | 1 | 1 | 0 | 0 |
| 5 days | 0 | 0 | 0 | 0 | 0 | 0 | 0 | 0 | 0 | 0 | 0 | 0 |

Gray shadow shows the days selected to calibrate the models for each time interval.

Workdays

| **Available days**  **of data** | **00-02** | **02-04** | **04-06** | **06-08** | **08-10** | **10-12** | **12-14** | **14-16** | **16-18** | **18-20** | **20-22** | **22-24** |
| --- | --- | --- | --- | --- | --- | --- | --- | --- | --- | --- | --- | --- |
| 1 day | 25 | 25 | 33 | 34 | 36 | 32 | 32 | 34 | 33 | 34 | 33 | 27 |
| 2 days | 16 | 15 | 25 | 29 | 30 | 30 | 29 | 32 | 30 | 32 | 30 | 19 |
| 3 days | 8 | 7 | 20 | 24 | 27 | 25 | 25 | 31 | 27 | 25 | 18 | 13 |
| 4 days | 5 | 6 | 13 | 18 | 20 | 22 | 21 | 23 | 24 | 22 | 13 | 10 |
| 5 days | 3 | 4 | 10 | 16 | 16 | 16 | 16 | 22 | 18 | 15 | 8 | 4 |
| 6 days | 2 | 2 | 8 | 11 | 10 | 11 | 12 | 13 | 16 | 14 | 6 | 3 |
| 7 days | 1 | 1 | 5 | 9 | 6 | 6 | 9 | 10 | 10 | 6 | 5 | 2 |
| 8 days | 0 | 0 | 3 | 6 | 2 | 2 | 4 | 3 | 2 | 3 | 1 | 1 |
| 9 days | 0 | 0 | 0 | 1 | 1 | 1 | 0 | 1 | 1 | 1 | 1 | 1 |
| 10 days | 0 | 0 | 0 | 0 | 0 | 0 | 0 | 1 | 1 | 1 | 1 | 1 |
| 11 days | 0 | 0 | 0 | 0 | 0 | 0 | 0 | 0 | 0 | 0 | 0 | 0 |

Gray shadow shows the days selected to calibrate the models for each time interval.
