# Supplementary material for: Modelling Terrestrial and Marine Foraging Habitats in Breeding Audouin's Gulls Larus audouinii: Timing Matters
Source: PLoS One. 2015 Apr 14;10(4):e0120799. doi: 10.1371/journal.pone.0120799 (PMC4397092; doi:10.1371/journal.pone.0120799)
Supplement: S3 Table — Percentage of lunar disk illuminated, and timing of moonrise, moonset, sunrise and sunset. Weekends are showed in light gray. Data from TPE. The Photographer’s Ephemeris. 2013. Software available http://photoephemeris.com/ (DOCX) [file pone.0120799.s008.docx]

**S3 Table. Moonlight information during the study period.** Percentage of lunar disk illuminated, and timing of moonrise, moonset, sunrise and sunset. Weekends are showed in light gray. Data from TPE. The Photographer’s Ephemeris. 2013. Software available <http://photoephemeris.com/>

| **Date** | **Day** | **illuminated lunar disk** | **moonrise** | **moonset** | **sunrise** | **sunset** |
| --- | --- | --- | --- | --- | --- | --- |
| 09/05/11 | Monday | 30.3% | 11:56 | 1:41 | 6:47 | 21:00 |
| 10/05/11 | Tuesday | 41.1% | 13:04 | 2:17 | 6:46 | 21:01 |
| 11/05/11 | Wednesday | 51.8% | 14:14 | 2:49 | 6:45 | 21:02 |
| 12/05/11 | Thursday | 63.1% | 15:25 | 3:20 | 6:44 | 21:03 |
| 13/05/11 | Friday | 73.9% | 16:38 | 3:50 | 6:43 | 21:04 |
| 14/05/11 | Saturday | 83.6% | 17:52 | 4:20 | 6:42 | 21:05 |
| 15/05/11 | Sunday | 91.4% | 19:07 | 4:54 | 6:41 | 21:06 |
| 16/05/11 | Monday | 96.9% | 20:23 | 5:32 | 6:40 | 21:07 |
| 17/05/11 | Tuesday | 100.0% | 21:35 | 6:17 | 6:39 | 21:08 |
| 18/05/11 | Wednesday | 99.6% | 22:40 | 7:09 | 6:38 | 21:09 |
| 19/05/11 | Thursday | 96.8% | 23:36 | 8:07 | 6:37 | 21:10 |
| 20/05/11 | Friday | 91.7% | 0:00 | 9:11 | 6:36 | 21:11 |
| 21/05/11 | Saturday | 84.7% | 0:23 | 10:16 | 6:35 | 21:12 |
| 22/05/11 | Sunday | 76.4% | 1:02 | 11:21 | 6:34 | 21:13 |
| 23/05/11 | Monday | 67.2% | 1:34 | 12:24 | 6:33 | 21:14 |
| 24/05/11 | Tuesday | 57.6% | 2:03 | 13:24 | 6:33 | 21:15 |
