# Supplementary material for: Modelling Terrestrial and Marine Foraging Habitats in Breeding Audouin's Gulls Larus audouinii: Timing Matters
Source: PLoS One. 2015 Apr 14;10(4):e0120799. doi: 10.1371/journal.pone.0120799 (PMC4397092; doi:10.1371/journal.pone.0120799)
Supplement: S4 Table — Spearman correlation matrix values for variable selection. In red are shown correlation coefficient values ǀrsǀ ≥0.70. *Variables eliminated prior to the modelling process. (DOCX) [file pone.0120799.s009.docx]

**S4 Table. Spearman correlation matrix.** Spearman correlation matrix values for variable selection. In red are shown correlation coefficient values ǀr_s_ǀ ≥0.70. *Variables eliminated prior to the modelling process.

|  | TrawDe06-08 | TrawDe08-10 | TrawDe10-12 | TrawDe12-14 | TrawDe14-16 | TrawDe16-18 | RiceFieldsCov* | MASL | RiceFieldsMASL | Bathymetry | PurSeDe00-02 | PurSeDe02-04 | PurSeDe04-06 | PurSeDe06-08 | PurSeDe08-10 | PurSeDe22-24 | Chl2011may | ColonyDist | ContinentDist* | CoastDist* | TrawPortDist* | PurSePortDist* | PortsDist | Slope* | PortsCov | SST2011may |
| --- | --- | --- | --- | --- | --- | --- | --- | --- | --- | --- | --- | --- | --- | --- | --- | --- | --- | --- | --- | --- | --- | --- | --- | --- | --- | --- |
| TrawDe06-08 | **1** | 0.59 | 0.57 | 0.60 | 0.75 | 0.59 | -0.25 | -0.47 | -0.22 | 0.00 | 0.45 | 0.43 | 0.50 | 0.49 | 0.36 | 0.48 | 0.38 | 0.19 | -0.17 | -0.05 | -0.42 | -0.35 | -0.34 | 0.22 | -0.03 | 0.30 |
| TrawDe08-10 | 0.59 | **1** | 0.78 | 0.83 | 0.70 | 0.30 | -0.24 | -0.45 | -0.22 | -0.04 | 0.30 | 0.30 | 0.36 | 0.29 | 0.26 | 0.30 | 0.16 | -0.05 | 0.10 | 0.15 | -0.15 | -0.02 | 0.02 | 0.04 | -0.05 | 0.26 |
| TrawDe10-12 | 0.57 | 0.78 | **1** | 0.81 | 0.62 | 0.32 | -0.24 | -0.45 | -0.21 | -0.09 | 0.30 | 0.30 | 0.35 | 0.29 | 0.26 | 0.30 | 0.16 | -0.01 | 0.15 | 0.17 | -0.10 | 0.03 | 0.07 | 0.10 | -0.05 | 0.24 |
| TrawDe12-14 | 0.60 | 0.83 | 0.81 | **1** | 0.73 | 0.33 | -0.24 | -0.45 | -0.21 | -0.06 | 0.32 | 0.31 | 0.35 | 0.30 | 0.28 | 0.31 | 0.19 | -0.03 | 0.11 | 0.15 | -0.15 | -0.02 | 0.01 | 0.07 | -0.05 | 0.26 |
| TrawDe14-16 | 0.75 | 0.70 | 0.62 | 0.73 | **1** | 0.55 | -0.24 | -0.45 | -0.22 | 0.02 | 0.33 | 0.34 | 0.38 | 0.36 | 0.30 | 0.37 | 0.30 | 0.01 | -0.05 | 0.04 | -0.33 | -0.21 | -0.20 | 0.08 | -0.03 | 0.26 |
| TrawDe16-18 | 0.59 | 0.30 | 0.32 | 0.33 | 0.55 | **1** | -0.31 | -0.58 | -0.28 | -0.03 | 0.44 | 0.42 | 0.45 | 0.53 | 0.50 | 0.56 | 0.51 | 0.09 | -0.07 | -0.05 | -0.22 | -0.26 | -0.22 | 0.24 | 0.01 | 0.28 |
| RiceFieldsCov* | -0.25 | -0.24 | -0.24 | -0.24 | -0.24 | -0.31 | **1** | 0.53 | 0.90 | 0.23 | -0.28 | -0.28 | -0.28 | -0.29 | -0.38 | -0.30 | -0.23 | -0.07 | -0.24 | -0.24 | -0.17 | 0.00 | -0.16 | -0.23 | -0.01 | -0.23 |
| MASL | -0.47 | -0.45 | -0.45 | -0.45 | -0.45 | -0.58 | 0.53 | **1** | 0.47 | 0.43 | -0.52 | -0.52 | -0.52 | -0.54 | -0.69 | -0.56 | -0.43 | 0.01 | -0.43 | -0.44 | -0.28 | -0.13 | -0.29 | -0.44 | 0.05 | -0.43 |
| RiceFieldsMASL | -0.22 | -0.22 | -0.21 | -0.21 | -0.22 | -0.28 | 0.90 | 0.47 | **1** | 0.21 | -0.25 | -0.25 | -0.25 | -0.26 | -0.35 | -0.27 | -0.21 | -0.06 | -0.22 | -0.22 | -0.15 | 0.00 | -0.15 | -0.21 | -0.01 | -0.21 |
| Bathymetry | 0.00 | -0.04 | -0.09 | -0.06 | 0.02 | -0.03 | 0.23 | 0.43 | 0.21 | **1** | 0.15 | 0.11 | 0.14 | 0.13 | -0.14 | 0.12 | 0.15 | -0.25 | -0.75 | -0.77 | -0.58 | -0.44 | -0.63 | -0.70 | 0.07 | -0.07 |
| PurSeDe00-02 | 0.45 | 0.30 | 0.30 | 0.32 | 0.33 | 0.44 | -0.28 | -0.52 | -0.25 | 0.15 | **1** | 0.83 | 0.80 | 0.78 | 0.58 | 0.73 | 0.54 | 0.08 | -0.23 | -0.19 | -0.22 | -0.39 | -0.32 | 0.12 | 0.00 | 0.16 |
| PurSeDe02-04 | 0.43 | 0.30 | 0.30 | 0.31 | 0.34 | 0.42 | -0.28 | -0.52 | -0.25 | 0.11 | 0.83 | **1** | 0.83 | 0.78 | 0.52 | 0.68 | 0.50 | 0.08 | -0.20 | -0.16 | -0.17 | -0.35 | -0.29 | 0.11 | -0.02 | 0.13 |
| PurSeDe04-06 | 0.50 | 0.36 | 0.35 | 0.35 | 0.38 | 0.45 | -0.28 | -0.52 | -0.25 | 0.14 | 0.80 | 0.83 | **1** | 0.83 | 0.55 | 0.68 | 0.49 | 0.05 | -0.21 | -0.17 | -0.20 | -0.39 | -0.31 | 0.10 | -0.03 | 0.15 |
| PurSeDe06-08 | 0.49 | 0.29 | 0.29 | 0.30 | 0.36 | 0.53 | -0.29 | -0.54 | -0.26 | 0.13 | 0.78 | 0.78 | 0.83 | **1** | 0.65 | 0.71 | 0.56 | 0.01 | -0.21 | -0.18 | -0.17 | -0.41 | -0.32 | 0.13 | -0.02 | 0.16 |
| PurSeDe08-10 | 0.36 | 0.26 | 0.26 | 0.28 | 0.30 | 0.50 | -0.38 | -0.69 | -0.35 | -0.14 | 0.58 | 0.52 | 0.55 | 0.65 | **1** | 0.57 | 0.51 | -0.06 | 0.10 | 0.11 | 0.14 | -0.13 | 0.00 | 0.31 | 0.00 | 0.28 |
| PurSeDe22-24 | 0.48 | 0.30 | 0.30 | 0.31 | 0.37 | 0.56 | -0.30 | -0.56 | -0.27 | 0.12 | 0.73 | 0.68 | 0.68 | 0.71 | 0.57 | **1** | 0.60 | 0.10 | -0.17 | -0.18 | -0.24 | -0.30 | -0.25 | 0.17 | 0.01 | 0.29 |
| Chl2011may | 0.38 | 0.16 | 0.16 | 0.19 | 0.30 | 0.51 | -0.23 | -0.43 | -0.21 | 0.15 | 0.54 | 0.50 | 0.49 | 0.56 | 0.51 | 0.60 | **1** | 0.04 | -0.39 | -0.24 | -0.27 | -0.41 | -0.38 | 0.28 | 0.02 | 0.03 |
| ColonyDist | 0.19 | -0.05 | -0.01 | -0.03 | 0.01 | 0.09 | -0.07 | 0.01 | -0.06 | -0.25 | 0.08 | 0.08 | 0.05 | 0.01 | -0.06 | 0.10 | 0.04 | **1** | -0.14 | -0.08 | -0.37 | -0.16 | -0.19 | 0.43 | 0.03 | 0.13 |
| ContinentDist* | -0.17 | 0.10 | 0.15 | 0.11 | -0.05 | -0.07 | -0.24 | -0.43 | -0.22 | -0.75 | -0.23 | -0.20 | -0.21 | -0.21 | 0.10 | -0.17 | -0.39 | -0.14 | **1** | 0.85 | 0.81 | 0.78 | 0.92 | 0.33 | -0.07 | 0.22 |
| CoastDist* | -0.05 | 0.15 | 0.17 | 0.15 | 0.04 | -0.05 | -0.24 | -0.44 | -0.22 | -0.77 | -0.19 | -0.16 | -0.17 | -0.18 | 0.11 | -0.18 | -0.24 | -0.08 | 0.85 | **1** | 0.68 | 0.61 | 0.77 | 0.38 | -0.07 | 0.12 |
| TrawPortDist* | -0.42 | -0.15 | -0.10 | -0.15 | -0.33 | -0.22 | -0.17 | -0.28 | -0.15 | -0.58 | -0.22 | -0.17 | -0.20 | -0.17 | 0.14 | -0.24 | -0.27 | -0.37 | 0.81 | 0.68 | **1** | 0.66 | 0.83 | 0.22 | -0.04 | -0.07 |
| PurSePortDist* | -0.35 | -0.02 | 0.03 | -0.02 | -0.21 | -0.26 | 0.00 | -0.13 | 0.00 | -0.44 | -0.39 | -0.35 | -0.39 | -0.41 | -0.13 | -0.30 | -0.41 | -0.16 | 0.78 | 0.61 | 0.66 | **1** | 0.86 | 0.11 | -0.05 | 0.22 |
| PortsDist | -0.34 | 0.02 | 0.07 | 0.01 | -0.20 | -0.22 | -0.16 | -0.29 | -0.15 | -0.63 | -0.32 | -0.29 | -0.31 | -0.32 | 0.00 | -0.25 | -0.38 | -0.19 | 0.92 | 0.77 | 0.83 | 0.86 | **1** | 0.23 | -0.06 | 0.15 |
| Slope* | 0.22 | 0.04 | 0.10 | 0.07 | 0.08 | 0.24 | -0.23 | -0.44 | -0.21 | -0.70 | 0.12 | 0.11 | 0.10 | 0.13 | 0.31 | 0.17 | 0.28 | 0.43 | 0.33 | 0.38 | 0.22 | 0.11 | 0.23 | **1** | -0.02 | 0.14 |
| PortsCov | -0.03 | -0.05 | -0.05 | -0.05 | -0.03 | 0.01 | -0.01 | 0.05 | -0.01 | 0.07 | 0.00 | -0.02 | -0.03 | -0.02 | 0.00 | 0.01 | 0.02 | 0.03 | -0.07 | -0.07 | -0.04 | -0.05 | -0.06 | -0.02 | **1** | 0.00 |
| SST2011may | 0.30 | 0.26 | 0.24 | 0.26 | 0.26 | 0.28 | -0.23 | -0.43 | -0.21 | -0.07 | 0.16 | 0.13 | 0.15 | 0.16 | 0.28 | 0.29 | 0.03 | 0.13 | 0.22 | 0.12 | -0.07 | 0.22 | 0.15 | 0.14 | 0.00 | **1** |
