# Supplementary material for: Modelling Terrestrial and Marine Foraging Habitats in Breeding Audouin's Gulls Larus audouinii: Timing Matters
Source: PLoS One. 2015 Apr 14;10(4):e0120799. doi: 10.1371/journal.pone.0120799 (PMC4397092; doi:10.1371/journal.pone.0120799)
Supplement: S1 Text — (DOCX) [file pone.0120799.s010.docx]

**S1 Text. Habitat use cover, Chl-a, and SST calculation.**

**Habitat use cover**

To convert this categorical variable to a continuous variable, we calculated the coating of habitat per pixel (0 to 1). This was done because of the BCN200 spatial resolution is greater than the working spatial resolution. The procedure wass as follows:

1. Polygons (rice fields or ports) were rasterized to a size less than 5 times the work spatial resolution (approximately coinciding with the Corine SR 100m). We used the same spatial origin in x, in this way the 25 pixels resulting overlapped with the work grid.
2. Using the ArcGis tool *focal statistics* and a window of 5x5 pixels, we calculated the average rice field cover (or ports) of 24 pixels adjacent to each of the pixels of the study area.
3. Finally the resulting raster was densified to the spatial resolution work by bilinear interpolation. In this way we obtained for each pixel the percentage of rice field (or ports) cover (Fig. S4).

**Chl-a and SST**

Both variables were downloaded from the Oceancolor web http://oceancolor.gsfc.nasa.gov/ (Feldman, 2012) in HDF format and level 2 processing (daily images). Level 2 was selected because of provides a spatial resolution of 1 km, unlike other downstream MODIS products, whose spatial resolution is 4 km (level 3). Thus we obtained the maximum resolution according to the working spatial resolution. The procedure was as follows:

1. All available images between 8 and 24 May 2011, both Chl-a (n = 14) and SST (n = 20), were downloaded (both Aqua and Terra) at Level 2
2. The georeferencing of these images was made ​​from the grid points of latitude and longitude provided in the HDF itself. For this we used ENVI software tool *Georeference MODIS*. We used for reprojection the nearest neighbor to not alter the radiometric image values​​
3. Once georeferenced, it was exported to MiraMon software. In the case of the Chl-a, NODATA values ​​corresponded to areas with clouds or land, so the selection of the information was very simple (in the absence of cloudless images for the study period, it was decided to make the average of available images)
4. For each image (day), a mask with data areas (without clouds) was done with MiraMon and assigned a value = 1. For each image, Nodata values have been reclassified to 0, and the sum of all this images, have been divided by the sum of the masks, to obtain the average
5. For SST images, clouds do not have Nodata value, but take the value of its temperature. Most of the clouds were removed automatically since they have values ​​outside the range of the sea temperature. In some cases these had to be removed by hand digitized masks. To average SST images obtained have proceeded in the same manner as for Chl-a. (Fig. S4).
