# Supplementary material for: Modelling Terrestrial and Marine Foraging Habitats in Breeding Audouin's Gulls Larus audouinii: Timing Matters
Source: PLoS One. 2015 Apr 14;10(4):e0120799. doi: 10.1371/journal.pone.0120799 (PMC4397092; doi:10.1371/journal.pone.0120799)
Supplement: S2 Text — (DOCX) [file pone.0120799.s011.docx]

**S2 Text. Environmental variables selection procedure.**

Once created environmental variables we assessed the potential correlation existing between them. For this aim we created a grid of points every 5 pixels and extracted the value of each variable using the ArcGIS tool *Extract Multi Values ​​to points*. With the data obtained we made ​​a Spearman correlation matrix in R software. We defined any pairs of variables with ǀr_s_ǀ ≥0.70 as highly correlated, and one of the variables was discarded for each such pair This way, six variables were eliminated. Fishing variables for different time intervals may be highly correlated between them, but were not eliminated since they would not be used simultaneously.
